# Beyond polyphagy and opportunism: natural prey of hunting spiders in the canopy of apple trees

**DOI:** 10.7717/peerj.9334

**Published:** 2020-06-19

**Authors:** László Mezőfi, Gábor Markó, Csaba Nagy, Dávid Korányi, Viktor Markó

**Affiliations:** 1 Department of Entomology, Szent István University, Budapest, Hungary; 2 Department of Plant Pathology, Szent István University, Budapest, Hungary; 3 Behavioural Ecology Group, Department of Systematic Zoology and Ecology, Eötvös Loránd University, Budapest, Hungary; 4 Research Institute for Fruitgrowing and Ornamentals, National Agricultural Research and Innovation Centre, Újfehértó, Hungary; 5 Institute of Ecology and Botany, “Lendület” Landscape and Conservation Ecology, Centre for Ecological Research, Vácrátót, Hungary; 6 GINOP Sustainable Ecosystems Group, Centre for Ecological Research, Tihany, Hungary

**Keywords:** Araneae, Beneficial arthropods, Natural diet, Intraguild predation, Food web, Trophic niche, Biocontrol potential, Ontogenetic shift, *Carrhotus xanthogramma*, *Philodromus cespitum*

## Abstract

Spiders (Araneae) form abundant and diverse assemblages in agroecosystems such as fruit orchards, and thus might have an important role as natural enemies of orchard pests. Although spiders are polyphagous and opportunistic predators in general, limited information exists on their natural prey at both species and community levels. Thus, the aim of this study was to assess the natural prey (realized trophic niche) of arboreal hunting spiders, their role in trophic webs and their biological control potential with direct observation of predation events in apple orchards. Hunting spiders with prey in their chelicerae were collected in the canopy of apple trees in organic apple orchards in Hungary during the growing seasons between 2013 and 2019 and both spiders and their prey were identified and measured. Among others, the composition of the actual (captured by spiders) and the potential (available in the canopy) prey was compared, trophic niche and food web metrics were calculated, and some morphological, dimensional data of the spider-prey pairs were analyzed. Species-specific differences in prey composition or pest control ability were also discussed. By analyzing a total of 878 prey items captured by spiders, we concluded that arboreal hunting spiders forage selectively and consume a large number of apple pests; however, spiders’ beneficial effects are greatly reduced by their high levels of intraguild predation and by a propensity to switch from pests to alternative prey. In this study, arboreal hunting spiders showed negative selectivity for pests, no selectivity for natural enemies and positive selectivity for neutral species. In the trophic web, the dominant hunting spider taxa/groups (*Carrhotus xanthogramma*, *Philodromus cespitum*, *Clubiona* spp., *Ebrechtella tricuspidata*, *Xysticus* spp. and ‘Other salticids’) exhibit different levels of predation on different prey groups and the trophic web’s structure changes depending on the time of year. Hunting spiders show a high functional redundancy in their predation, but contrary to their polyphagous nature, the examined spider taxa showed differences in their natural diet, exhibited a certain degree of prey specialization and selected prey by size and taxonomic identity. Guilds (such as stalkers, ambushers and foliage runners) did not consistently predict either prey composition or predation selectivity of arboreal hunting spider species. From the economic standpoint, *Ph. cespitum* and *Clubiona* spp. were found to be the most effective natural enemies of apple pests, especially of aphids. Finally, the trophic niche width of *C. xanthogramma* and *Ph. cespitum* increased during ontogeny, resulting in a shift in their predation. These results demonstrate how specific generalist predators can differ from each other in aspects of their predation ecology even within a relatively narrow taxonomic group.

## Introduction

Spiders play an important role in ecosystems as predators of various invertebrate groups. In certain habitats, according to the highest realistic estimates, spiders might kill up to approximately 200 kg prey ha^−1^ year^−1^ (Nyffeler, 2000), which by extrapolation suggests that the global spider community might consume up to 400–800 million tons of prey annually (Nyffeler & Birkhofer, 2017). Generally, spiders are regarded as polyphagous (preying on a wide variety of prey) and opportunistic (taking their prey as a function of each prey species’ availability), although some degree of selectivity in foraging is often observed (Nentwig, 1980; Whitney et al., 2018; Eitzinger et al., 2019). Moreover, stenophagy has evolved in certain groups of spiders, e.g. myrmecophagy in Zodariidae or araneophagy in Salticidae (Pekár, Coddington & Blackledge, 2012; Pekár & Toft, 2015). Spiders mainly prey on insects, of which the preferred size is primarily ~50–80% of the spiders’ size (Nentwig & Wissel, 1986; Foelix, 2011), but they can also feed on other invertebrates (Nyffeler & Symondson, 2001; Nyffeler et al., 2017) or vertebrates (Nyffeler & Knörnschild, 2013; Nyffeler & Pusey, 2014; Nyffeler, Edwards & Krysko, 2017), eggs of various arthropods (Nyffeler et al., 1990; Ahmed et al., 2018) or even on plant nectar and pollen (Nyffeler, 2016; Nyffeler, Olson & Symondson, 2016).

In agroecosystems, spiders can contribute significantly to pest control by consuming a large number of various insect pests (Nyffeler & Benz, 1988; Young & Edwards, 1990; Marc, Canard & Ysnel, 1999; Nyffeler & Sunderland, 2003; Birkhofer et al., 2008; Liu et al., 2015; Suenaga & Hamamura, 2015; Lefebvre et al., 2017). A recent meta-analysis (Michalko et al., 2019) of 58 studies found that spiders suppressed agricultural insect pests in 79% of the cases, although their efficacy varied among crops. From an economic point of view, hunting spiders have special importance, as they collect their prey directly from the surface of the crop and thus they more frequently consume less mobile stages (e.g., eggs, larvae, nymphs) of various arthropods than web-building spiders (Marc, Canard & Ysnel, 1999; Nyffeler, 1999). Also, hunting spiders have a wider trophic niche compared to web-builders (Nyffeler, 1999; Michalko & Pekár, 2016). Furthermore, besides direct predation, hunting spiders have several other non-consumptive effects on pests/herbivores (Mansour, Rosen & Shulov, 1981; Sunderland, 1999; Beleznai et al., 2015; Bucher, Menzel & Entling, 2015; Tholt et al., 2018) and due to the consumptive and non-consumptive effects, hunting spiders can also improve crop performance indirectly (Schmitz, Beckerman & O’Brien, 1997; Schmitz & Suttle, 2001; Schmitz, 2008).

Trophic niche width (or diet breadth) of spider species varies along a continuum from extremely narrow (feeding on a single prey taxon) to extremely wide (feeding on all available prey taxa) diet range, although some differences may exist even at the more polyphagic end of the continuum (Pekár, Coddington & Blackledge, 2012; Pekár & Toft, 2015). Although these generalist predators can effectively reduce pest numbers (Symondson, Sunderland & Greenstone, 2002; Nyffeler & Sunderland, 2003), many factors can influence their role in pest suppression and food-web dynamics at both community and individual levels (Michalko, Pekár & Entling, 2019). Several environmental factors and functional traits can be directly or indirectly involved, including the presence or absence of alternative prey (Madsen, Terkildsen & Toft, 2004; Kuusk & Ekbom, 2010), the intensity of intraguild predation or predator interference (Snyder & Wise, 1999; Wise, 2006; Petráková et al., 2016; Michalko et al., 2017), the season of the year (Snyder & Wise, 2001), hunting strategy or guild (Schmitz, 2008; Miller, Ament & Schmitz, 2014; Liu et al., 2015; Michalko & Pekár, 2016), or ontogenetic differences (Bartos, 2011).

It is hard to assess what spiders’ diets consist of or what role various species play in food webs or trophic cascades. In laboratory experiments, spiders might accept more prey types than in their natural environment (Líznarová & Pekár, 2019) and thus, these studies provide only limited insight into the natural diet of spiders (Greenstone, 1999). In the field, there are many methods to obtain information about the realized trophic niche of invertebrate predators (Sunderland, 1988; Symondson, 2002; Birkhofer et al., 2017; Macías-Hernández et al., 2018). Although they are labor-intensive, direct in situ observations can provide the most reliable data about the natural diet of a focal predator species (Greenstone, 1999; Birkhofer et al., 2017; Pekár, García & Viera, 2017), so it is not surprising that this method is widely used to assess diet concerning different species of spiders (Yeargan, 1975; Lockley & Young, 1987; Morse, 1981; Nyffeler, Dean & Sterling, 1992; Huseynov, 2005, 2007). Many studies on the natural diet of spiders have focused on web-building spiders because the observation of sedentary species is easier than tracking mobile hunters, and in case of web-builders there is an opportunity to collect prey carcasses from their web. In many studies investigating hunting spiders, the diet of only one species or species-pair was examined without comparing the composition of potential and actual prey (see the references in Pekár, Coddington & Blackledge (2012), Michalko & Pekár (2016) and Pekár, García & Viera (2017)). Thus, very little is known about the field diet of hunting spider assemblages, especially in the canopy layer, and limited information is available on how actual prey relates to potential prey, and how one species’ diet relates to another.

Spiders form abundant and diverse assemblages in apple orchards and can contribute to the suppression of various apple pests (Bogya, Szinetár & Markó, 1999; Bogya, Markó & Szinetár, 2000; Michalko & Pekár, 2015; Lefebvre et al., 2017), although their function in biological control has been less studied in orchards (Michalko et al., 2019). In the light of the above, the aim of this study was to assess the natural prey (realized trophic niche) of arboreal hunting spiders, their role in trophic webs, and their biological control potential using direct observation of predation events in apple orchards. More specifically, our objectives were (1) to evaluate the natural prey and (2) predation selectivity of the hunting spider assemblage in the canopy of apple trees, (3) to compare the preferred prey of the most abundant hunting spider species or groups (4) concerning their hunting guild. We also aimed to determine how (5) size and (6) the life stage of hunting spiders affect the composition and size of their prey.

## Materials and Methods

### Data collection

Data on the natural diet (actual prey) of the arboreal hunting spider assemblage was collected between 2013 and 2019 in apple orchards in Hungary. For this, apple trees were visually inspected regularly in organic orchards, and hunting spiders with prey in their chelicerae were collected during the growing season (from the beginning of April to the end of October). The vast majority of the observations (*N* = 788, almost 90% of the data) came from one organic apple orchard located at Újfehértó (an experimental orchard of the Research Institute for Fruitgrowing and Ornamentals, National Agricultural Research and Innovation Centre), in Szabolcs-Szatmár-Bereg County, eastern Hungary. A further 37, 31 and 22 observations on the hunting spiders’ natural prey (for a total of 878 observations) were collected in apple orchards of the Szent István University in the vicinity of Újfehértó (Szabolcs-Szatmár-Bereg County), in Pest County and Bács-Kiskun County, respectively. The orchard located in Újfehértó (~3.3 ha, 47°49′11.5″N, 21°39′56.9″E) was planted on flat land, on a fine sandy soil in autumn 2002 and contained the cultivars ‘Florina’, ‘Prima’, ‘Rajka’, ‘Releika’, ‘Rewena’, ‘Rubinola’ and ‘Topaz’ on ‘M9’ and ‘Remo’ and ‘Resi’ on ‘M26’ rootstocks. It had 32 rows, each consisting of ~90–135 trees. Rows were spaced 5 m apart and apple trees were spaced 1.5 and 2.25 m apart within rows. The orchard was surrounded by other orchards (cherry, apple) as well as other agricultural areas.

Our in situ observations were conducted both day and night (approximate ratio 7:3) to get information not only on the prey of the diurnal hunting spiders but also on the nocturnal ones. Apple trees were examined mainly between 9:00 and 12:00, between 14:00 and 18:00 and between 20:00 and 23:00 (after sunset). Spiders with prey in their chelicerae were collected (with a glass vial) and the prey was taken from the spiders to prevent any further degradation. In some cases, just the prey was collected because the spider escaped or because we did not want to influence other trials conducted in the orchard. After collecting the spiders with their prey, the material was taken to the laboratory of the Department of Entomology, Szent István University (Budapest, Hungary), and both the spider and the prey were identified (with a binocular stereo microscope, Leica MZ6) to the lowest taxonomic level possible. Moreover, in spiders, the width of the prosoma and in case of the preys (if their conditions allowed) the width of the thorax were measured with 0.1 mm accuracy using an ocular micrometer calibrated with a stage micrometer. In juvenile spiders where the species-level identification was not possible (e.g., in *Philodromus* species), spiders were raised to the adult stage (on *Drosophila hydei* Sturtevant) in the laboratory. Spiders were identified after Nentwig et al. (2019) and the taxonomic names follow the nomenclature of the WSC (2019). The spiders were stored in 70% ethanol, while the prey items were stored mainly dry in glass vials. Approximately 4–5% of the prey items collected were unidentifiable due to the high level of degradation and were excluded from the analyses. The dataset (see Data S1) contains only the cases where both the spider and its prey were identifiable (878 observations).

To obtain information on the potential prey community of arboreal hunting spiders, a D-VAC sampler was used. In the organic apple orchard located at Újfehértó, suction samples were taken at monthly intervals between April and October in 2016 and 2017 (on 14 sampling dates). On each sampling date, five samples were taken. Each sample consisted of suction samples collected from one (left or right) side of the canopy of four randomly selected apple trees in a randomly selected row. For the samplings, a ~25 cm long, tapering gauze bag (mesh < 0.5 mm) was inserted into the 12 cm diameter intake nozzle of the D-VAC sampler. Suction sampling was carried out during dry weather, approximately between 9:00 and 14:00. The collected material was sorted and identified (mainly to order, suborder, family or genus level) in the laboratory.

### Preparation of data for analysis

For most analyses, the spiders were classified into six groups: (1) *Carrhotus xanthogramma* (Latreille), (2) Other salticids, (3) *Philodromus cespitum* (Walckenaer), (4) *Ebrechtella tricuspidata* (Fabricius), (5) *Xysticus* spp. s. lat. and (6) *Clubiona* spp. The main criterion for group formation was that the number of records in a particular group should exceed 5% of the total sample (i.e., a group must contain at least 44 observations) at the lowest possible taxonomic level. Thus, while *C. xanthogramma*, *Ph. cespitum* and *E. tricuspidata* were collected in sufficiently large numbers (44 < *n*) for analyses, the other species had to be placed in genus- (*Xysticus* spp. and *Clubiona* spp.) or family-level (Other salticids) groups. The group ‘Other salticids’ comprises the data on other spider species belonging to the family Salticidae, mainly three species: (1) *Heliophanus auratus* C. L. Koch, (2) *H. cupreus* (Walckenaer), (3) *Salticus scenicus* (Clerck), but not including *C. xanthogramma*. The group ‘*Xysticus* spp. s. lat.’ (hereafter *Xysticus* spp.) comprises the following seven species: (1) *Xysticus acerbus* Thorell, (2) *X. cristatus* (Clerck), (3) *X. kochi* Thorell, (4) *X. lanio* C. L. Koch, (5) *X. striatipes* L. Koch (currently *Spiracme striatipes*, see Breitling, 2019), (6) *X. ulmi* (Hahn) and (7) unidentified juveniles of *Xysticus* spp. Finally, the group ‘*Clubiona* spp.’ consists of *C. frutetorum* L. Koch and unidentified juveniles of *Clubiona* spp. (Tables S1 and S2). To compare the hunting strategies of the species collected, they were classified using two different guild classification systems (Uetz, Halaj & Cady, 1999; Cardoso et al., 2011). According to Uetz, Halaj & Cady (1999), hunting spider guilds included (1) stalkers (*C. xanthogramma* and Other salticids), (2) ambushers (*Ph. cespitum*, *E. tricuspidata* and *Xysticus* spp.) and (3) foliage runners (*Clubiona* spp.). Based on a more recent guild classification by Cardoso et al. (2011) our hunting spider groups could be grouped into just two guilds: ambush hunters (*E. tricuspidata* and *Xysticus* spp.) and other hunters (*C. xanthogramma*, Other salticids, *Ph. cespitum* and *Clubiona* spp.).

Using the slightly modified prey classification system of Michalko & Pekár (2015), prey items retrieved from spiders or collected by D-VAC sampling were classified into the following 16 taxonomic groups: Acari, Araneae, Coleoptera, Lepidoptera, Formicidae, Other (non-formicid) Hymenoptera, Brachycera, Nematocera (i.e., all non-Brachycera dipterans), Auchenorrhyncha, Heteroptera, Sternorrhyncha, Ephemeroptera, Neuroptera, Psocoptera, Thysanoptera and Trichoptera. The prey categories that had relative abundances of less than 1% in the total actual prey of the whole arboreal hunting spider assemblage, namely Acari, Ephemeroptera, Neuroptera, Psocoptera, Thysanoptera and Trichoptera, were pooled into the group of ‘Other prey’ in certain statistical analyses.

To evaluate the biological control potential of the hunting spiders, the prey items were categorized according to their economic status in apple orchards in Central Europe as pests, natural enemies and neutral arthropod groups. A prey species was considered to be a pest if at least one of its life stages is known to feed on any parts of the apple tree. The pest category included some beetles (mainly weevils), some moths (both adult and larva of for example, leaf miners, tortrix moths), some leafhoppers and planthoppers, lace bugs and all aphid and psyllid (Sternorrhyncha) species. Natural enemies are defined as species that can feed (at least in one of their life stages) on any stage of arthropods that were previously categorized as pests. This category includes red velvet mites (Trombidiidae), spiders, predatory beetles (e.g., coccinellids, carabids), parasitoid wasps, hoverflies, zoophagous bugs (e.g., some mirids and anthocorids) and lacewings. Finally, the neutral category was comprised of other (non-pest and non-natural enemy) prey species. For prey that could be identified only to suborder, such as Nematocera, the classification was made according to the dominant characteristics of the taxon (i.e., the vast majority of Nematocera occurring in apple orchard are neutral species (Alford, 2014)).

Only a few species of Diptera cause damage on apple in Europe (*Dasineura mali* (Keifer), *Resseliella oculiperda* (Rübsaamen) (Nematocera) and *Phytomyza heringiana* Hendel (Brachycera)) and they are of minor importance (Alford, 2014). None of these species or their damage were found in the orchard (Újfehértó). *Drosophila suzukii* (Matsumura) (and other *Drosophila* species, Brachycera) can breed exclusively on overripe, bruised and rotten apples, and usually infests fallen fruits. Therefore, in apple orchards, this species considered to be as a decomposer (Alford, 2014). Based on these considerations, Nematocera and Brachycera dipterans (excluding hoverflies) were classified as neutral species. The role of ants could change seasonally depending on the size of the aphid colonies. In apple orchards, ants act as mutualists in the early phase of the aphid population development (Nagy, Cross & Markó, 2015). However, later when the aphid abundance is already high, ants follow rather than drive aphid abundances (Markó et al., 2013). Because of the above criteria, and because of vast majority of ants captured by spiders were dispersing males or workers at the peak of aphid abundance, ants were also classified as neutral prey. Economic classification can be seen in Data S1 in more detail.

### Data analysis

All statistical analyses were performed within the R (v.3.5.3.) statistical environment (R Core Team, 2019). For all analyses, the natural prey data were pooled across orchards and years (see later), except for the comparison of actual and potential prey where only the data collected at the same place (Újfehértó) and in the same years (2016–2017) were analyzed.

### Comparison of actual versus potential prey

Generalised Linear Mixed Models with binomial error structure (GLMM-b) (Pekár & Brabec, 2016) were used to compare the relative frequency of prey taxa or economic groups between the actual and potential prey. For these analyses, abundance data in each prey category were pooled for each season (spring, summer and fall) regarding the given year (2016 or 2017). In the model, the response variable was a matrix (Pekár & Brabec, 2016) containing the abovementioned seasonal counts of a given prey category, and the difference of these seasonal counts and the seasonal sums of all actual or potential prey counts for the given year. Prey taxa, their economic status, prey type (actual vs. potential), season, and year were entered in the model as fixed factors and the interactions were calculated (Prey taxa/Economic status × Prey type, Prey taxa/Economic status × Season and Prey taxa/Economic status × Year). The model included an observation level random effect to avoid overdispersion. Significant interaction (e.g., between Prey taxa and Prey type) implies that hunting spiders select prey disproportionately. Model contrasts for the prey types within each prey categories were computed separately using the R package “emmeans” (Lenth et al., 2020). For the raw data of the previous analyses, see Data S2.

To assess the degree of selectivity shown by spiders, Ivlev’s electivity indices (IE) were computed based on the relative abundances of the actual and potential prey categories (collected in Újfehértó 2016–2017). To filter out the effect of year, first, the index values were calculated for each year and prey category (taxonomic or economic), and then mean index values were calculated. For economic categories, the abundances were calculated as sums of all prey items belonging to the given category. The IE values range between −1 and +1, where negative and positive values indicate negative and positive prey selection relative to prey availability in the environment, respectively (Cock, 1978).

### Comparison of spider and prey composition on temporal and spatial scale

The accuracy of our hand sampling method was evaluated and the selectivity in spider predation was examined from another perspective as well. For this, a Mantel test based on Morisita dissimilarity distance was performed to calculate the correlation between the matrices of monthly abundance of the six spider groups in hand-collected versus suction samples (Újfehértó, 2016/2017, see Table S3) and between the matrices of monthly abundance of actual versus potential prey groups (Újfehértó, 2016/2017, see Table S4) using the “vegdist” function of the R package “vegan” (Oksanen et al., 2019) and the “mantel” function of the R package “ecodist” (Goslee & Urban, 2019). The same method was used to compare the abundances of the actual prey groups for the six most abundant spider taxa in Újfehértó, 2016/2017 with those from other sites or years (see Table S5). For further statistics, the data from Újfehértó 2016/2017 were pooled with the rest of the observations (as noted in “Results”).

### Food web metrics, niche width and niche overlap

To compare the trophic characteristics of the six most abundant spider taxa, specialization metrics (a measure of stenophagy), trophic niche width, and degree of niche overlap were calculated based on the taxonomic composition of the spiders’ prey (see the upper part of the Table S6). Food web specialization was calculated at both the network and species level (Blüthgen, Menzel & Blüthgen, 2006). Web specialization (*H*_2_′) was calculated using “H2fun” function, while species-level specialization (*d′*) was calculated for the six spider taxa using the “dfun” function of the R package “bipartite” (Dormann, Fruend & Gruber, 2020). The values of these quantitative, frequency-based specialization metrics range from 0 to 1, where 0 corresponds to total generalization and 1 corresponds to extreme specialization (Blüthgen, Menzel & Blüthgen, 2006). To estimate the trophic niche breadth Levins’ index (*B*) (Krebs, 1999) was calculated using the R package “spaa” (Zhang, 2016). Niche overlap indices (NOs) between the six spider taxa based on prey taxonomic composition (categorical data) and prey size (log-transformed continuous data) were calculated using the script provided by Geange et al. (2011). Null models with 10,000 permutations were used for each niche dimension (taxonomic, size and overall) to test the possible differences between the occupied niches of the hunting spider groups (Geange et al., 2011). Bonferroni correction was used to avoid the errors of multiple comparisons. 1 – NOs as a distance measures were used to visualize differences among spider groups in their niches with multidimensional scaling (MDS) (Geange et al., 2011) using “monoMDS” function of the R package “vegan” (Oksanen et al., 2019). Levins’ *B* ranges from 1 to *n*, where *n* is the total number of resource states and 1 corresponds with maximum specialization, while NO ranges from 0 to 1 where 1 corresponds with complete overlap (Krebs, 1999; Geange et al., 2011). As prey availability data were not available for every year and orchard, they were not involved in the above-mentioned measures.

### Comparison of prey composition of spider groups using fourth-corner analysis

The “fourth-corner analysis” is an adequate multivariate technique for testing relationships between abundance data and species or other environmental trait matrices (Dray & Legendre, 2008; Brown et al., 2014; Comay & Dayan, 2018). The analysis provides coefficients indicating the strength of the association between each pair of traits and tests for significance using a permutation test (Dray & Legendre, 2008; Brown et al., 2014). In the analyses, the response variable was the abundance of the observed prey taxa, while the environmental components included species composition of the predators (i.e., spider groups) and seasonality (i.e., spring, summer and fall). To calculate these coefficients, a LASSO-penalised regression model assuming a negative binomial distribution for model errors was applied by the “mvabund” R package (Wang et al., 2012). The size of a coefficient reflects the relative importance of the given interaction, that is, how a coefficient changes the slope of the relationship between abundance and a given environmental variable. A spider group (or season) and a prey taxon was considered as either negatively or positively associated if the absolute value of the coefficient was greater than 0.03.

### Analyses of the predator–prey size relationship and the variation in body size

To analyze the spider-prey size relationship, GLMs with gamma error structure and log-link (GLM-g) were used due to the Gamma distribution of the prey thorax widths (Michalko & Pekár, 2015; Pekár & Brabec, 2016). As a new variable, the thorax-prosoma (i.e., prey–predator) size ratio (prey thorax width divided by spider prosoma width) was computed in each possible spider-prey pair (if data was available). After that, the variation of the taxa-specific body traits, the predator prosoma width, prey thorax width and thorax-prosoma ratio were analyzed separately by Linear Models (LMs). In all models, the body trait was the response variable, while the spider group, season and prey taxa were entered as predictor variables. The body size variables were log-transformed to approach normal distribution. Special attention was paid to the life-stage-specific differences in two spider species (*C. xanthogramma* and *Ph. cespitum*), given both species were collected in large numbers. Within both species, the individuals were grouped into two life-stages, juveniles and adults (i.e., adult and subadult individuals). In adults, the data of different sexes were pooled for both species, because there were no sex-specific differences in the investigated morphological traits (i.e., prey thorax width: *C. xanthogramma*: *t* = −0.315, df = 75.406, *P* = 0.753; *Ph. cespitum*: *t* = 1.646, df = 45.803, *P* = 0.107; thorax-prosoma ratio: *C. xanthogramma*: *t* = −0.878, df = 65.904, *P* = 0.383; *Ph. cespitum*: *t* = 0.973, df = 39.225, *P* = 0.337). The following model structure was run in both species, separately: the log-transformed thorax-prosoma ratio was the response variable, while the Life-stage (i.e., juvenile and adult), Prey taxa and Season (spring, summer and fall) were the predictor variables (as factors). For testing the post-hoc contrasts, Welch’s t-test with the Holm’s correction (to avoid the errors of multiple comparisons) was used.

To compare the widths of the niches with respect to prey size between the different arboreal hunting spider groups or between the life stages of *C. xanthogramma* and *Ph. cespitum*, the variances (*S*^*2*^) in prey–predator size ratios were computed (as Michalko & Pekár, 2015). To compare variances, Levene’s test was used. This test tolerates a slight deviation from a normal distribution (Reiczigel, Harnos & Solymosi, 2018), and thus, the data were not transformed to avoid the false interpretation of the results, because transformation might have affected the variability of our data (Feng et al., 2014). In the case of multiple comparisons, the *P*-values of Levene’s test were adjusted using Holm’s correction.

## Results

**Figure 1:**
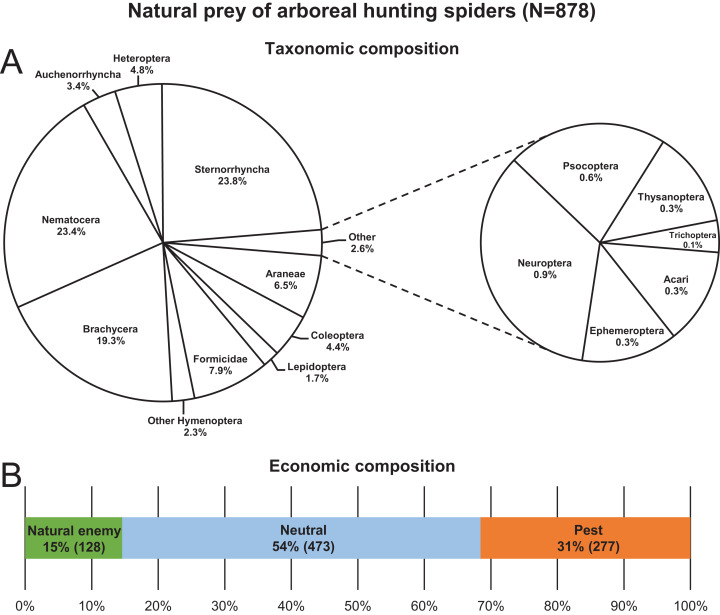
Natural prey (*N* = 878) of arboreal hunting spiders collected in apple orchards. Taxonomic (A) and economic (B) composition.

A total of 878 hunting spider individuals, belonging to 29 species and seven families, were collected with identifiable prey in their chelicerae from the canopy of apple trees between 2013 and 2019 (Table S1). The most abundant spider taxa/groups in decreasing order were *C. xanthogramma*, *Ph. cespitum*, *Clubiona* spp., Other salticids, *E. tricuspidata* and *Xysticus* spp., which accounted for 89% of all spiders in the dataset (Table S2). Approximately 0.8–1.2 spiders with a prey item in the chelicerae were collected per person-hour, and 34, 46 and 20% of the individuals were collected in the morning, afternoon and after sunset, respectively. Species of Sternorrhyncha, Brachycera and Nematocera together accounted for 66.5% of the total prey of the hunting spider assemblage, and spiders most frequently (54%) preyed upon arthropods that were irrelevant to pest management (neutral prey) (Fig. 1; Table S7). Aphids and spiders were preyed upon to the greatest extent within the pest and natural enemy groups, respectively (Tables S8 and S9). In contrast, none of the hunting spiders collected in this study preyed on larvae or adults of codling moth (*Cydia pomonella* (L.)) the key pest of apple in Europe. Two salticid individuals (one each of *C. xanthogramma* and *Heliophanus* sp.) were observed to feed on lacewing eggs. For the monthly abundance count of spider and prey groups see Tables S3 and S4. For the total hunting spider assemblage, prey size was significantly related to spider size (GLM-g, *F*_1, 647_ = 235.74, *P* < 0.001, *R*^2^ = 0.23), with the average prey thorax width and spider prosoma width being 1.13 and 1.72 mm, respectively, while the average prey–predator size ratio was 0.67 (SD: 0.34) (see Fig. S1).

### Comparison of actual and potential prey

**Figure 2:**
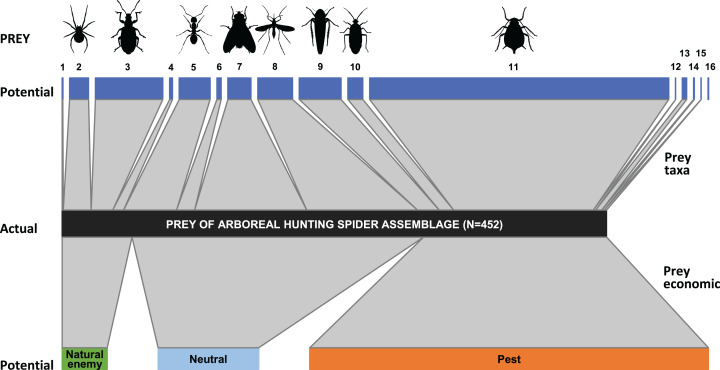
Trophic link structure for the arboreal hunting spider assemblage (middle bar) and its prey (upper and lower bars) at Újfehértó, Hungary, 2016–2017. Trapezoids connecting the bars show the frequency of prey categories in the natural diet of the spider assemblage (actual prey, *N* = 452; center of connector) and in the canopy of apple trees (potential prey, *N* = 11,421; upper and lower end of connectors). Non‐parallel sides in a trapezoid suggest selectivity in spiders predation on the focal prey category, with an outward tapering trapezoid suggesting an overrepresentation and an outward widening trapezoid suggesting underrepresentation of the given taxon or economic group in the diet of spiders. Note that the figure based on the 2 years sum of actual and potential prey items. Numbers refer to following prey taxa: (1) Acari, (2) Araneae, (3) Coleoptera, (4) Lepidoptera, (5) Formicidae, (6) Other Hymenoptera, (7) Brachycera, (8) Nematocera, (9) Auchenorrhyncha, (10) Heteroptera, (11) Sternorrhyncha, (12) Ephemeroptera, (13) Neuroptera, (14) Psocoptera, (15) Thysanoptera, (16) Other prey items.

**Figure 3:**
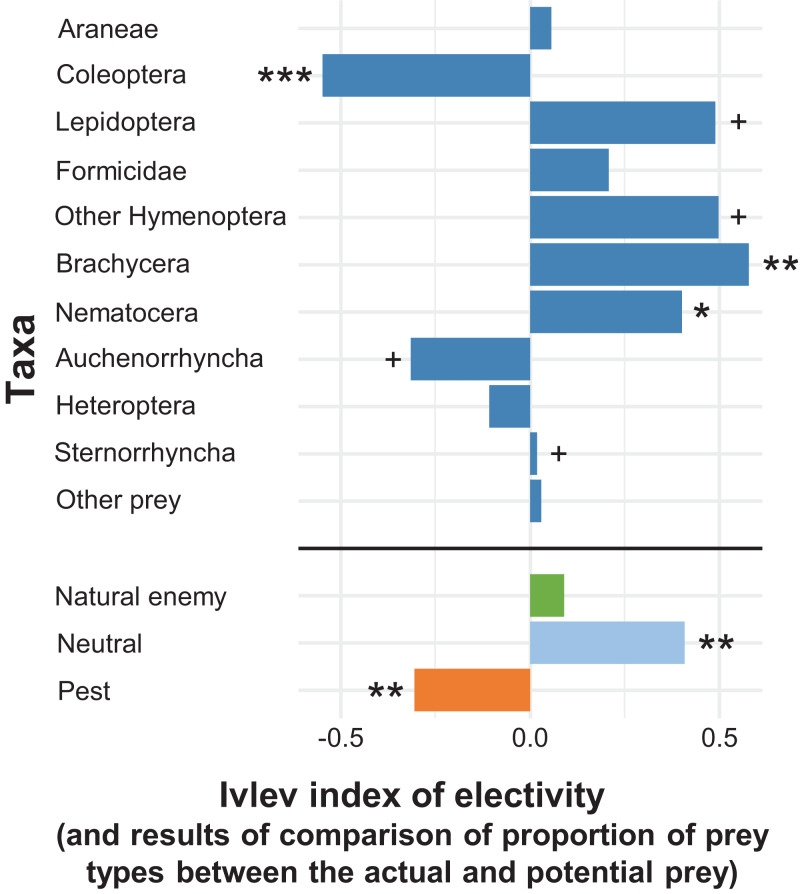
Ivlev’s electivity index for the arboreal hunting spider assemblage, Újfehértó, Hungary, 2016–2017. A total of 2-year means of the index values. In the given group asterisks indicate significant (*P < 0.05, **P < 0.01, ***P < 0.001) or marginally significant (^+^*P* < 0.1) deviation between spider diet and relative abundance of potential prey based on model contrasts. For the indices and *P* values see Table S10.

In the apple orchard located in Újfehértó in 2016 and 2017 the seasonal composition of hand-collected spiders (with prey) and suction-sampled spiders correlated (Mantel’s *r* = 0.605, *P* = 0.004), which showed that our hand-collected sample represented well the total hunting spider assemblage in the canopy. However, the Mantel test showed no correlation between the seasonal composition of actual (held in the chelicerae) and potential (suction-sampled) prey groups (*r* = 0.013, *P* = 0.957) (for the raw matrices, see Tables S3 and S4). This suggests that the composition of actual prey was not strongly driven by the composition of potential prey. In accordance with this finding, the relative frequencies of actual prey groups differed significantly from those of potential prey (GLMM-b, Prey taxa vs. Prey type (actual vs. potential) interaction: LRT_10_ = 37.680, *P* < 0.001), demonstrating that hunting spiders, as a community, showed selectivity in their diet. Brachycera and Nematocera were captured significantly more (GLMM-b, contrasts, *P* = 0.002 and *P* = 0.025, respectively), while Coleoptera was captured significantly less (GLMM-b, contrast, *P* < 0.001) frequently than their abundance would suggest (Figs. 2 and 3; Table S10). Lepidoptera, Other Hymenoptera and Sternorrhyncha were marginally significantly, positively selected and Auchenorrhyncha was marginally significantly, negatively selected by spiders, while Araneae, Formicidae, Heteroptera and Other prey taxa were preyed proportionally to their availability (Figs. 2 and 3, for GLMM-b contrasts, see Table S10). Brachycera had the highest Ivlev’s index value, while Coleoptera had the lowest value of the Ivlev’s index being our measure of selective predation (Fig. 3; Table S10). The relative frequencies of prey groups differed significantly among seasons (GLMM-b, Prey taxa × Season interaction: LRT_20_ = 83.765, *P* < 0.001), but not among years (GLMM-b, Prey taxa × Year interaction: LRT_10_ = 12.846, *P* = 0.232).

**Figure 4:**
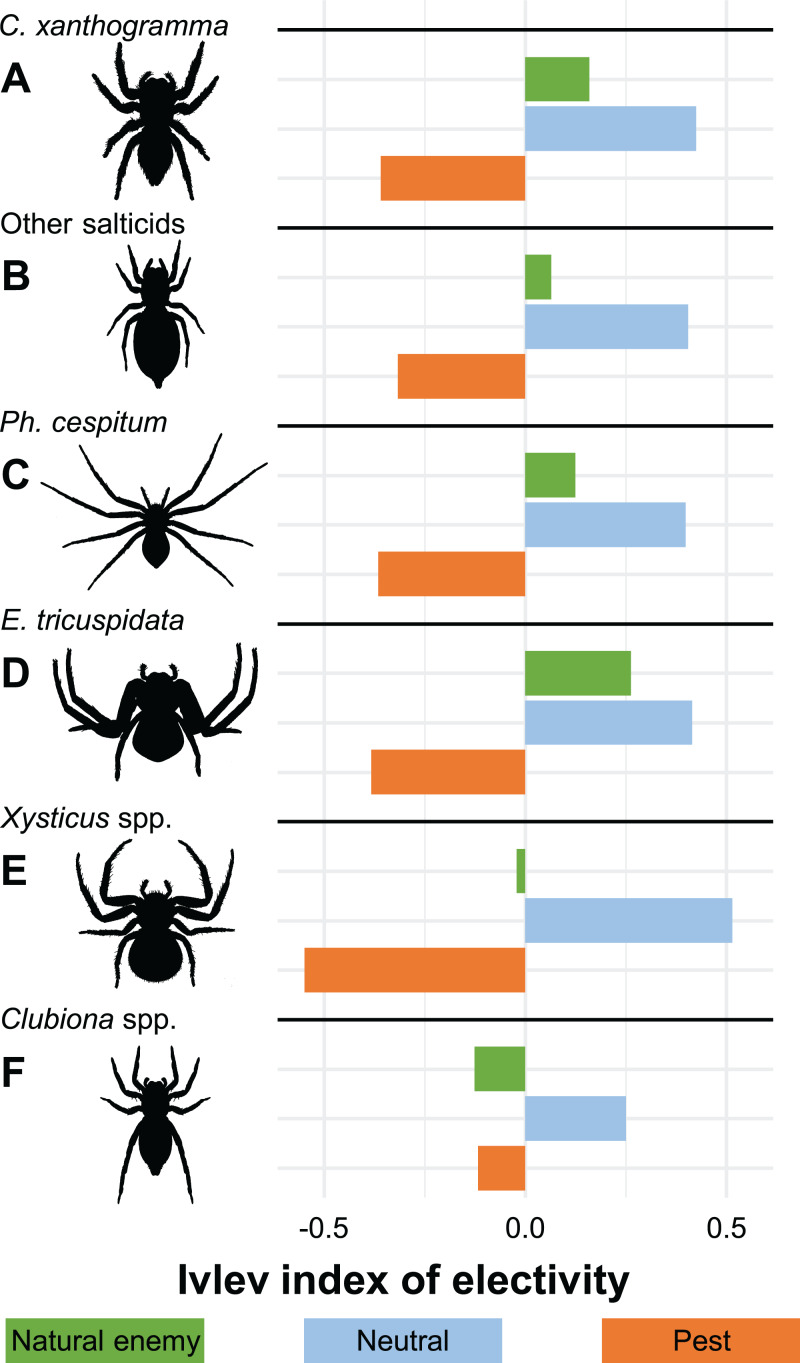
Ivlev’s electivity index for arboreal hunting spider groups, Újfehértó, Hungary, 2016–2017. A total of 2-year means of the index values. *N* = 214, 26, 73, 24, 22 and 57 for (A) *C. xanthogramma*, (B) Other salticids, (C) *Ph. cespitum*, (D) *E. tricuspidata*, (E) *Xysticus* spp. and (F) *Clubiona* spp., respectively.

Considering the economic status of prey species, the proportions of the categories in the actual prey differed significantly from the potential prey (GLMM-b, Prey status × Prey type (actual vs. potential) interaction: LRT_2_ = 15.286, *P* < 0.001, Figs. 2 and 3; Table S10). We found that the actual prey of arboreal hunting spiders consisted of proportionally more neutral prey, and fewer pest individuals (GLMM-b, both contrast *P* = 0.002), as compared with the relative abundance of potential prey (Fig. 3; Table S10). Natural enemies were preyed proportionally to their availability (Fig. 3; Table S10). The diets of all hunting spider groups show a similar pattern (Fig. 4). Based on the Ivlev’s index, four out of the six spider taxa selected natural enemies positively (Fig. 4). The proportions of the economic categories differed marginally between seasons (GLMM-b, Prey status × Season interaction: LRT_4_ = 9.427, *P* = 0.051) without difference between years (GLMM-b, Prey status × Year interaction: LRT_2_ = 3.284, *P* = 0.194).

### Food web metrics, niche width and niche overlap

**Figure 5:**
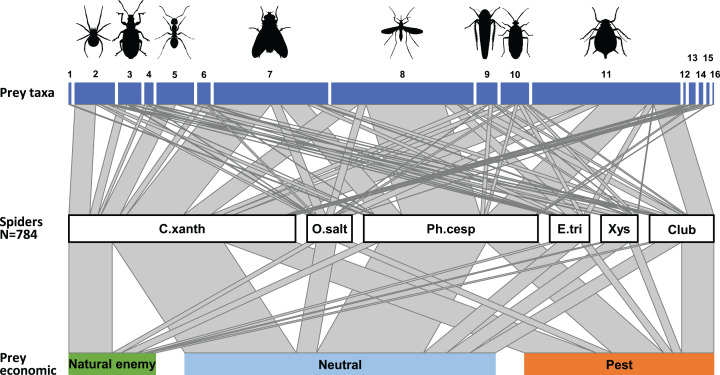
Trophic interactions between the most abundant hunting spider groups and the arthropod community in the canopy of apple trees. Whole growing season, *N* = 784. The middle bars represent spider groups and upper and bottom bars represent the spiders’ prey divided taxonomically and according their economic status. The width of the links between the trophic levels depict the frequency of interactions and bar widths indicate the relative abundance of each category. Numbers refer to following prey taxa: (1) Acari, (2) Araneae, (3) Coleoptera, (4) Lepidoptera, (5) Formicidae, (6) Other Hymenoptera, (7) Brachycera, (8) Nematocera, (9) Auchenorrhyncha, (10) Heteroptera, (11) Sternorrhyncha, (12) Ephemeroptera, (13) Neuroptera, (14) Psocoptera, (15) Thysanoptera, (16) Trichoptera; Spiders: *C. xanth* = *Carrhotus xanthogramma*; *O. salt* = Other salticids; *Ph. cesp* = *Philodromus cespitum*; *E. tri* = *Ebrechtella tricuspidata*; *Xys* = *Xysticus* spp.; *Club* = *Clubiona* spp.

For further analyses, we focused only on the most abundant hunting spider groups. As the abundances of the actual prey groups for the six most abundant spider taxa at Újfehértó, 2016/2017 were correlated (Mantel’s *r* = 0.515, *P* = 0.003) with those from other sites or years (see Table S5), the data were pooled across all sites and years. Figure 5 shows the trophic interactions between the spider groups and the canopy-dwelling arthropod community for the whole growing season. Overall, considering each group’s abundance, the highest predation pressure for most prey groups (Araneae, Formicidae, Other Hymenoptera, Brachycera, Auchenorrhyncha, Heteroptera and Sternorrhyncha) was imposed by *C. xanthogramma*. Most nematoceran prey were consumed by *Ph. cespitum*, while coleopterans were preyed on mainly by *Xysticus* spp. In addition, *Ph. cespitum* and *Clubiona* spp. exerted a high predation pressure on Sternorrhyncha, and *Xysticus* spp. did so on Formicidae (Fig. 5). The majority of natural enemies were consumed by *C. xanthogramma*, and the diets of *Ph. cespitum* and *Clubiona* spp. had the highest number of pests relative to the number of captured natural enemies (Fig. 5, for the raw data of the food-web, see Table S6). The seasonal abundance of the spider and potential prey groups, and therefore the food web structure, showed significant seasonal change (Figs. S2–S4). While *Ph. cespitum* was the most abundant hunting spider species in spring, *C. xanthogramma* dominated in summer and fall. Brachycera, Nematocera, and Sternorrhyncha were the most abundant prey groups in spring, summer and fall, respectively (Figs. S2–S4).

Food web specialization (*H*_2_′) was the highest in spring and the lowest in summer (Table 1). In general, hunting spiders were found to be generalists as their species-level specialization (*d′*) was low (values are mostly close to 0) and their trophic niche breadth (*B*) was wide. *Xysticus* spp., followed by *Ph. cespitum*, was the most specialized (most stenophagous) group and, in accordance with this, had the narrowest niche breadth (Table 1).

**Table 1:** Trophic niche width (*B*) and specialization metrics (*d′*and *H*_2_′) for hunting spider groups.

	*Carrhotus xanthogramma*	Other salticids	*Philodromus cespitum*	*Ebrechtella tricuspidata*	*Xysticus* spp.	*Clubiona* spp.
Levins’ niche breadth (*B*)						
Whole season	6.512	5.158	3.275	5.231	3.842	4.183
Predator specialization (*d*′)*						
Whole season	0.074	0.045	0.186	0.065	0.397	0.066
Spring	0.262	0.075	0.211	0.048	0.582	0.186
Summer	0.043	0.157	0.139	0.094	0.326	0.060
Fall	0.142	0.161	0.200	0.212	0.449	0.097
Food web specialization (*H*_2_′)*						
Whole season	0.142					
Spring	0.256					
Summer	0.130					
Fall	0.190					

**Note:**

* Specialization indices range from 0 for extreme generalization to 1 for extreme specialization.

**Figure 6:**
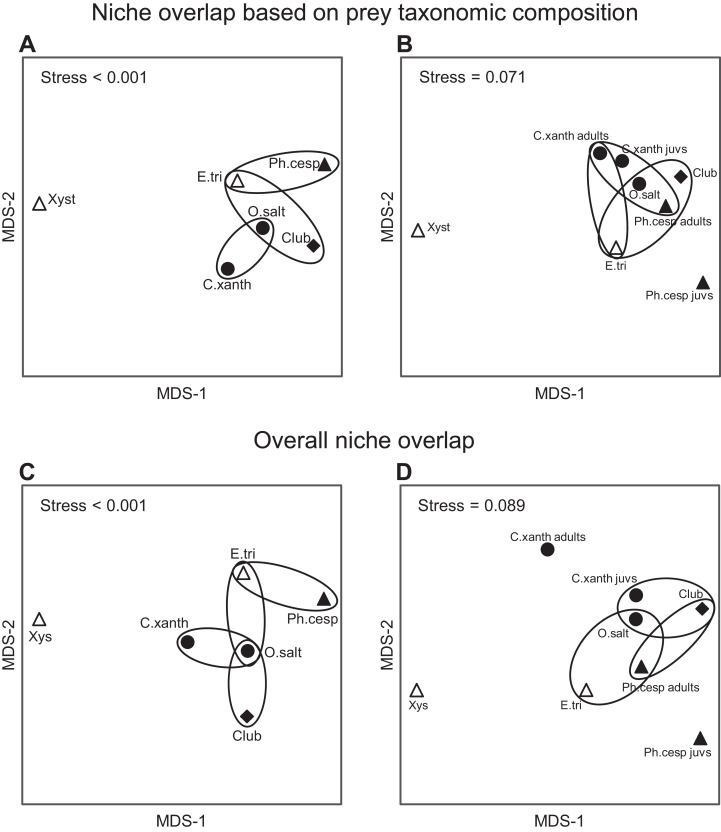
Trophic niche overlap between the most abundant arboreal hunting spider groups in apple orchards. Interspecific similarities in niche overlap based on taxonomic composition of spiders’ natural prey (A and B) and on two functional traits (C and D): (1) taxonomic composition of natural prey and (2) prey size. (A and C) the six most abundant spider groups; (B and D) the same but *C. xanthogramma* and *Ph. cespitum* were split to juveniles (all juvenile stages) and adults (subadults and adults). Similarities are represented graphically as multi-dimensional scaling. Ellipses encircle species occupying niches that were not identified as significantly different using null model tests. Different marks and fill indicate different guilds: circle, stalkers; triangle, ambushers; square, foliage runners (based on guild classification by Uetz, Halaj & Cady (1999)); empty marks, ambush hunters; solid marks, other hunters (based on guild classification by Cardoso et al. (2011)). Spiders: *C. xanth* = *Carrhotus xanthogramma*; *O. salt* = Other salticids; *Ph. cesp* = *Philodromus cespitum*; *E. tri* = *Ebrechtella tricuspidata*; *Xys* = *Xysticus* spp.; *Club* = *Clubiona* spp.

Considering the taxonomic composition of their prey, spider groups exhibited a relatively high level of trophic niche overlap (0.61 < NO in all comparisons), except for *Xysticus* spp., which had a relatively distinct prey composition (NO < 0.39 in all comparisons) (Fig. 6A; Table S11). The highest levels of niche overlap (0.73 ≤ NO) were observed between the following group pairs: *C. xanthogramma* and Other salticids, *Clubiona* spp. and Other salticids, and *E. tricuspidata* and Other salticids (Fig. 6A; Table S11). Spider groups displayed significant clustering in their distribution across niche space with respect to taxonomic composition of their prey (null model: 10,000 permutations, *P* < 0.001, Fig. 6A; Table S11). In contrast, spider groups exhibited a high level of niche overlap (0.72 ≤ NO in all comparisons) without significant clustering across niche space with respect to size of their prey (null model: 10,000 permutations, *P* = 0.135, Table S11). When we considered both niche dimensions combined (taxonomic identity and prey size), the realized niches of the spider groups clustered in niche space (null model: 10,000 permutations, *P* < 0.001, Fig. 6C; Table S11). Although the overall niche overlap between spider groups remained relatively high (between 0.51 and 0.85, Table S11), we found significant differences in 11 out of 15 pairwise comparisons (Fig. 6C). For the detailed NOs and pairwise comparisons see Tables S11 and S12.

### Fourth-corner analysis of spider-prey associations

**Figure 7:**
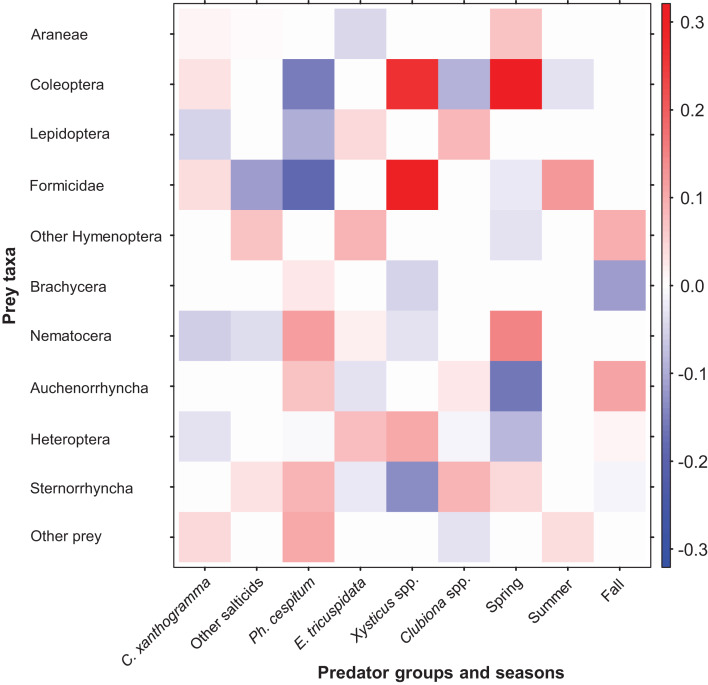
Fourth-corner analysis, including standardized coefficients of prey taxa vs. spider groups and seasonal predictors (GLM model-based approach with LASSO penalty). Darker colors indicate stronger associations than paler ones; positive associations are indicated by red, negative associations are indicated by blue color. For the coefficients see Table S13.

Fourth-corner analysis revealed that the variables Spider groups (GLM-nb, Dev_12, 5_ = 148.95, *P* = 0.01) and Season (GLM-nb, Dev_10, 2_ = 82.01, *P* = 0.005) significantly contributed to the prey selection by spiders. Furthermore, the interaction between Spider groups and Season also explained a significant amount of variance in prey abundance (GLM-nb, Dev_0, 10_ = 216.97, *P* = 0.001). Coefficients for the significant predictors of prey abundance are depicted in Fig. 7 and the exact values are shown in Table S13. Prey taxa varied in their abundance across spider groups and within the growing season. Taking into account the differences in total and seasonal abundances we found that, as compared to the other spiders, *C. xanthogramma* was positively associated (PA) with the prey groups Formicidae (almost exclusively winged males) and Coleoptera and negatively associated (NA) with Nematocera, Lepidoptera and Heteroptera. Similar selectivity was observed in other spider groups as well: Other salticids (PA, Other Hymenoptera, Sternorrhyncha; NA, Formicidae, Nematocera), *Ph. cespitum* (PA, Nematocera, Sternorrhyncha, Auchenorrhyncha; NA, Formicidae, Coleoptera, Lepidoptera), *E. tricuspidata* (PA, Other Hymenoptera, Heteroptera, Lepidoptera; NA, Araneae, Auchenorrhyncha), *Xysticus* spp. (PA, Formicidae, Coleoptera, Heteroptera; NA, Sternorrhyncha, Brachycera, Nematocera), *Clubiona* spp. (PA, Sternorrhyncha, Lepidoptera; NA, Coleoptera) (Fig. 7). The coefficient matrix also indicates significant seasonal variation in predation of certain prey taxa (e.g., Araneae, Coleoptera, Nematocera, Auchenorrhyncha) throughout the season (Fig. 7).

### Intraguild differences and interguild similarities

Based on the guild classification of Uetz, Halaj & Cady (1999), marked intraguild differences (*Ph. cespitum* vs. *Xysticus* spp. and *E. tricuspidata* vs. *Xysticus* spp. (null model: 10,000 permutations, *P* < 0.001 in both comparisons)) and high interguild similarities (*E. tricuspidata* vs. Other salticids, Other salticids vs. *Clubiona* spp. and *E. tricuspidata* vs. *Clubiona* spp.) were found in the composition (taxonomic or taxonomic + size) of natural prey (Figs. 5 and 6; Table S11). Prey preferences could also differ within a guild (e.g., in ambushers (*Ph. cespitum* vs. *E. tricuspidata* vs. *Xysticus* spp.), Fig. 7). Based on the guild classification of Cardoso et al. (2011), certain species also showed significant differences in their diet (*Ph. cespitum* vs. *Clubiona* spp., *C. xanthogramma* vs. *Ph. cespitum*, *C. xanthogramma* vs. *Clubiona* spp. and *E. tricuspidata* vs. *Xysticus* spp. (null model: 10,000 permutations, *P* < 0.001 in all comparisons, Fig. 6; Table S11)) or preferences (e.g., *C. xanthogramma* vs. *Ph. cespitum* or *E. tricuspidata* vs. *Xysticus* spp., respectively, Fig. 7) within the guilds of other hunters or ambush hunters. Furthermore, despite belonging to different guilds, *E. tricuspidata* and Other salticids or *E. tricuspidata* and *Ph. cespitum* showed no difference in trophic niche occupancy (Fig. 6; Table S11).

### Predator–prey size relationships

**Figure 8:**
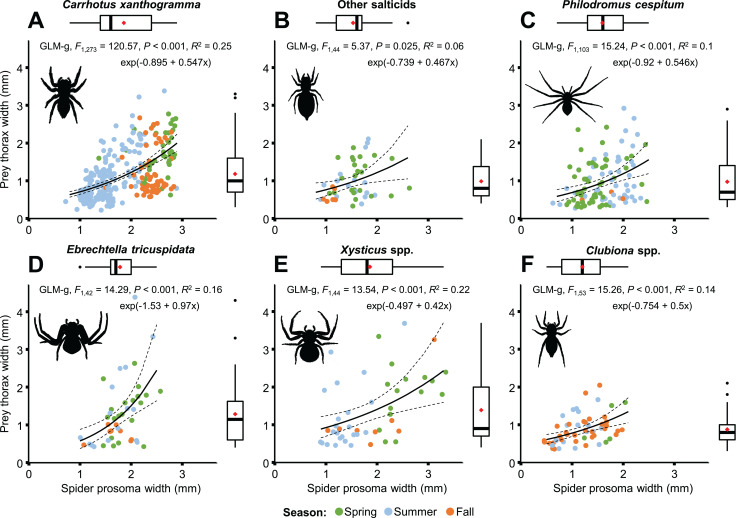
Relationship between spider and prey size (spider prosoma and prey thorax widths, jittered) for the most abundant arboreal hunting spider groups (A–F) in apple orchards. On the marginal boxplots red squares indicate means, see Table 2.

**Figure 9:**
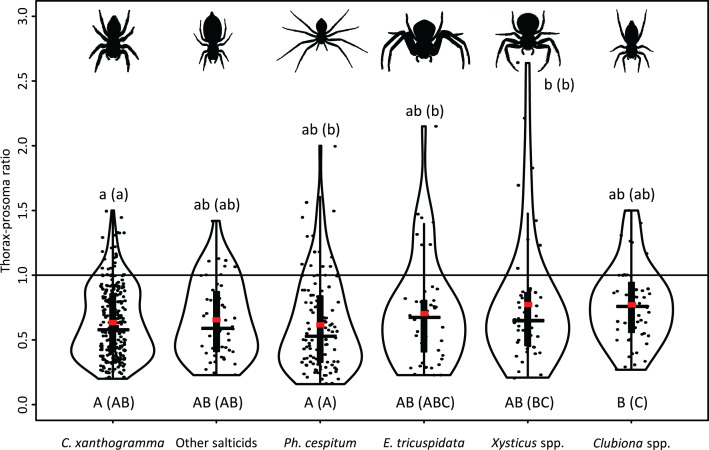
Prey-spider (prey thorax vs. spider prosoma) size ratios (jittered) for the arboreal hunting spider groups. Red square—mean; black horizontal solid line—median; black vertical rectangle—interquartile range. Different capital letters indicate significant differences among means, while different lowercase letters indicate significant differences among variances at *P* < 0.05 level. Letters in parentheses refer to pairwise comparisons with unadjusted *P* values.

A moderately strong exponential relationship was found between the spider and prey size for all six hunting spider groups (Fig. 8). Spider size differed between spider groups (*F*_5, 553_ = 31.543, *P* < 0.001), seasons (*F*_2, 553_ = 67.337, *P* < 0.001), and also between prey taxa (*F*_10, 553_ = 8.703, *P* < 0.001). On average, *C. xanthogramma* and *Xysticus* spp. had the widest, while *Clubiona* spp. had the narrowest prosoma (Table 2). Prey size differed between spider groups (*F*_5, 553_ = 8.499, *P* < 0.001), seasons (*F*_2, 553_ = 20.554, *P* < 0.001) and between prey taxa (*F*_10, 553_ = 30.946, *P* < 0.001). *Xysticus* spp. had prey with the widest, while *Clubiona* spp. had prey with the narrowest thorax (Table 2). The thorax-prosoma ratio differed among the spider groups (*F*_5, 553_ = 5.014, *P* < 0.001), among the seasons (*F*_2, 553_ = 13.176, *P* < 0.001) and among the different prey groups (*F*_10, 553_ = 24.222, *P* < 0.001). Compared to their own size, *Ph. cespitum* and *C. xanthogramma* caught the smallest whereas *Xysticus* and *Clubiona* spp. caught the relatively largest prey items and the difference between the first two species and *Clubiona* spp. was significant (Fig. 9; Table 2). Furthermore, *C. xanthogramma* differed significantly from *Xysticus* spp. in niche width with respect to prey size (*S*^*2*^ of thorax-prosoma ratios, Fig. 9).

**Table 2:** Spider prosoma and prey thorax widths, and thorax-prosoma ratios (mean ± SD) for hunting spider groups and seasons.

Spider taxa						Pooled	Season		
*Carrhotus xanthogramma*	Other salticids	*Philodromus cespitum*	*Ebrechtella tricuspidata*	*Xysticus* spp.	*Clubiona* spp.		Spring	Summer	Fall
N*									
275	46	105	44	46	55	571	161	293	117
Spider prosoma width (mm)									
1.86 (0.56) C	1.53 (0.36) B	1.59 (0.42) B	1.78 (0.35) C	1.86 (0.68) BC	1.20 (0.43) A	1.71 (0.55)	1.92 (0.59) b	1.53 (0.39) a	1.89 (0.66) b
Prey thorax width (mm)									
1.18 (0.64) B	0.99 (0.49) AB	0.97 (0.61) A	1.28 (0.85) AB	1.39 (0.88) B	0.88 (0.39) A	1.12 (0.66)	1.30 (0.69) b	1.06 (0.66) a	1.03 (0.56) a
Thorax-prosoma ratio									
0.64 (0.28) A	0.66 (0.29) AB	0.62 (0.36) A	0.70 (0.41) AB	0.77 (0.50) AB	0.77 (0.30) B	0.66 (0.33)	0.68 (0.33) ab	0.69 (0.35) b	0.59 (0.27) a

**Notes:**

* Spiders with no prosoma or prey thorax width data were excluded.

Different capital letters indicate significant differences between spider groups, while different lowercase letters indicate significant differences between seasons at *P* < 0.05 level.

**Figure 10:**
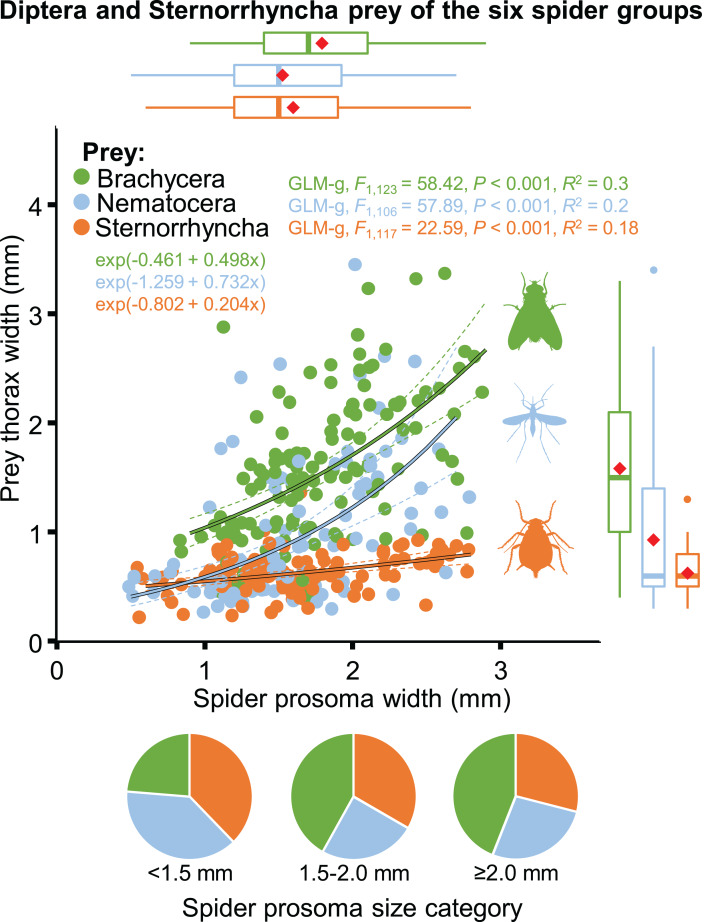
Relationship between spider and prey size (prosoma and thorax widths, jittered, *N* = 352) for the arboreal hunting spider groups and their main prey taxa, Brachycera, Nematocera and Sternorrhyncha. On the marginal boxplots red squares indicate the mean. Pie charts show the relative frequency of the three main prey groups for three different spider size categories (*N* = 135, 117 and 100, respectively).

The size of spiders and prey items decreased from spring to summer. Spider size increased afterwards whereas prey size remained low (Table 2; Fig. S5). As a consequence, the thorax-prosoma ratio was identical in spring and summer (*P* = 0.834) and decreased in fall (compared to spring, *P* = 0.054; or summer, *P* = 0.020) (Table 2; Fig. S5). Analysed separately, the prey size was significantly related to spider size for all three main prey groups (Brachycera, Nematocera, Sternorrhyncha) (Fig. 10). However, the prey–predator (thorax-prosoma) size ratio was significantly different (*P* < 0.001 in all comparisons), with Brachycera being the largest prey caught by a same-sized spider, indicating that the taxonomic identity of the prey influenced the prey–predator ratio (Fig. 10).

### Life stages of *C. xanthogramma* and *Ph. cespitum*

Only *C. xanthogramma* and *Ph. cespitum* were collected in numbers high enough to analyze their prey in more detail. Comparing the prey of the spider life stages, the following results were obtained: *C. xanthogramma* adults had the widest trophic niche breadth (*B* = 6.76), followed by *C. xanthogramma* juveniles (*B* = 5.85), *Ph. cespitum* adults (*B* = 4.32) and *Ph. cespitum* juveniles (*B* = 2.51) (Table S14). Considering the taxonomic composition of their prey (Table S15), these four groups showed a high level of niche overlap (0.70 < NO) except for the lower overlap (NO < 0.54) between *Ph. cespitum* juveniles and both adults and juveniles of *C. xanthogramma* (Fig. 6B; Table S12). When *C. xanthogramma* and *Ph. cespitum* were split to juveniles and adults, spider groups displayed significant clustering across niche space regarding taxonomic composition (null model: 10,000 permutations, *P* < 0.001, Fig. 6B) and size of the prey (null model: 10,000 permutations, *P* = 0.008) and across niche space incorporating these two functional traits (null model: 10,000 permutations, *P* < 0.001, Fig. 6D). *Ph. cespitum* adults occupied a trophic niche different from that of juvenile conspecifics (null model: 10,000 permutations, *P* = 0.001), indicating an ontogenetic niche shift (Fig. 6B). Taking into account both niche dimensions (taxonomic identity and prey size), adults and juveniles of both species differed from each other in niche occupancy (*C. xanthogramma*: null model: 10,000 permutations, *P* < 0.001; *Ph. cespitum*: null model: 10,000 permutations, *P* = 0.001; Fig. 6D; Table S12).

**Figure 11:**
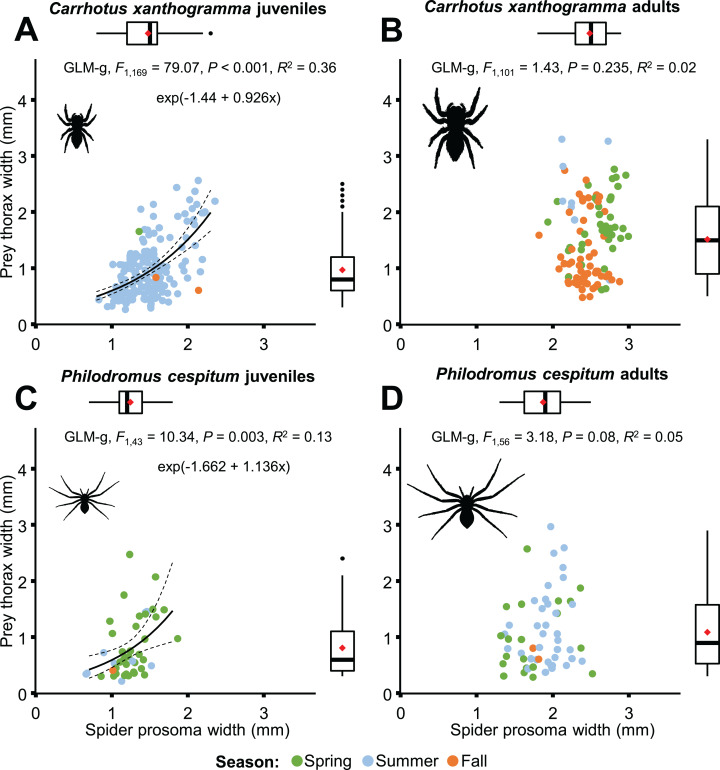
Relationship between spider prosoma and prey thorax widths (jittered) for juveniles and adults of *Carrhotus xanthogramma* (A and B) and *Philodromus cespitum* (C and D). Adults (B and D) comprise both subadult and adult individuals. On the marginal boxplots red squares indicate the mean values.

Although prey size was significantly related to spider size in both species (Fig. 8), different results were obtained when life stages were taken into consideration (Fig. 11). A significant relationship was found between predator and prey size for juveniles, but not for adults (Fig. 11). However, the thorax-prosoma size ratio was similar for the two life stage groups (*C. xanthogramma*: *F*_1, 260_ = 2.814, *P* = 0.095; *Ph. cespitum*: *F*_1, 91_ = 1.288, *P* = 0.259; Fig. S6), while it was different among the various prey groups (*C. xanthogramma*: *F*_10, 260_ = 24.133, *P* < 0.001; *Ph. cespitum*: *F*_8, 91_ = 6.338, *P* < 0.001). The three most abundant prey groups (Brachycera, Nematocera, Sternorrhyncha) differed from each other: (1) *C. xanthogramma*: Brachycera vs. Nematocera (*P* < 0.001), Brachycera vs. Sternorrhyncha (*P* < 0.001), Nematocera vs. Sternorrhyncha (*P* = 0.001) and (2) *Ph. cespitum*: Brachycera vs. Nematocera (*P* < 0.001), Brachycera vs. Sternorrhyncha (*P* < 0.001), Nematocera vs. Sternorrhyncha (*P* = 0.019) in relation to thorax-prosoma ratio, that is, a spider of a given size mostly caught larger Brachycera prey items than those of Nematocera or Sternorrhyncha (Fig. S7). Among seasons, the size ratio was different for *C. xanthogramma* (*F*_2, 260_ = 9.776, *P* < 0.001) but not for *Ph. cespitum* (*F*_2, 91_ = 0.358, *P* = 0.7).

We found that *C. xanthogramma* adults had numerically lower size variance, while *Ph. cespitum* adults had significantly greater variance (*S*^*2*^) in their size (prosoma width) compared to that in juveniles (Levene’s tests: *C. xanthogramma*: *F*_1, 272_ = 3.319, *P* = 0.07; *Ph. cespitum*: *F*_1, 101_ = 5.2, *P* = 0.025). Adults of both species had significantly greater variance in the size of their prey (thorax width) than juveniles (Levene’s tests: *C. xanthogramma*: *F*_1, 277_ = 19.282, *P* < 0.001; *Ph. cespitum*: *F*_1, 181_ = 6.745, *P* = 0.01). However, the variance of thorax-prosoma ratio (niche width with respect to prey size) was not different between the life stages (Levene’s tests: *C. xanthogramma*: *F*_1, 272_ = 0.166, *P* = 0.685; *Ph. cespitum*: *F*_1, 101_ = 0.252, *P* = 0.617). For detailed data see Table S14.

## Discussion

Based on 878 predator-prey records, we analyzed the prey composition, biological control potential, and trophic interactions of arboreal hunting spiders in apple orchards. Although they were found to be polyphagous predators in general, hunting spiders selectively preyed upon canopy arthropods and the different spider species/groups differed from each other either in their diet composition or their prey size selection.

### Prey composition, selectivity and efficiency in biological control

Two-thirds of the hunting spiders’ prey were Sternorrhyncha, Brachycera or Nematocera (Fig. 1). Interestingly, these are also the main prey groups of the web-building spiders in agricultural ecosystems (Birkhofer et al., 2018) and are the groups most often consumed by spiders in general (Birkhofer & Wolters, 2012; Michalko & Pekár, 2016). Most Sternorrhyncha prey were aphids (mainly *Dysaphis plantaginea* (Passerini) and *Aphis pomi* De Geer). Although aphids are regarded as a low-quality food (Toft, 1995, 2005; Bilde & Toft, 2001), they appear relatively frequently among the prey of spiders (Alderweireldt, 1994; Harwood, Sunderland & Symondson, 2005; Kerzicnik et al., 2012). What is more, hunting spiders can contribute to aphid suppression in various agricultural habitats (Birkhofer et al., 2008; De Roincé et al., 2013; Lefebvre et al., 2017). Ants (mainly *Lasius niger* (L.)) made up almost 8% of the prey of hunting spiders and comprised of both ant workers and winged males/queens. The fifth most frequent prey taxon was Araneae (e.g., theridiid or linyphiid males or other hunting spiders such as *C. xanthogramma*, *Ph. cespitum*), which comprised 6.5% of the total prey (Fig. 1). Intraguild predation is very common among hunting spiders (Hodge, 1999; Birkhofer & Wolters, 2012; Mestre et al., 2013) and spiders can make up to a quarter or a third of the hunting spiders’ diet (Michalko & Pekár, 2016). Furthermore, in this study, we observed two unusual predation events: a *C. xanthogramma* and a *Heliophanus* sp. that both fed on stalked chrysopid eggs. Oophagy, although uncommon, has been found in salticids (Nyffeler et al., 1990; Ahmed et al., 2018), and predation on lepidopteran eggs by *C. xanthogramma* was observed by Hirose et al. (1980).

Some prey types were consumed significantly more or less often by the arboreal hunting spiders than expected from their respective abundances in the environment (Figs. 2 and 3; Tables S7 and S10), indicating that these spiders are not strict opportunists and do not feed in a frequency-dependent manner. In general, the groups Brachycera and Nematocera (and possibly Other Hymenoptera, Lepidoptera and Sternorrhyncha) were overrepresented, while Coleoptera (and possibly Auchenorrhyncha) were underrepresented in the actual prey of the hunting spider community. Selective foraging was found both in epigeal (e.g., lycosids) and arboreal (e.g., philodromids) hunting spiders (Michalko & Pekár, 2015; Whitney et al., 2018; Eitzinger et al., 2019).

Hunting spiders preyed mostly (54% of the diet) on arthropods irrelevant to pest management in apple orchards in Central Europe such as Brachycera (excluding hoverflies), Nematocera and Formicidae (Fig. 1). They also consumed a significant number (31%) of pests, e.g., aphids, *Phyllobius* spp. (Coleoptera: Curculionidae), *Metcalfa pruinosa* (Say) (Auchenorrhyncha: Flatidae), and psyllids, (Table S8) although some of these apple-feeding arthropods are considered only minor pests of apple in Hungary (e.g., *M. pruinosa*). The rest of the prey (15% of the diet) was made up of natural enemies such as spiders, zoophagous bugs, parasitic wasps, hoverflies and lacewings (Table S9).

The hunting spider assemblage showed the highest selectivity (positive preference) for neutral prey species, and spiders preyed on pests less than would be expected based on their availability in the canopy of apple trees (Figs. 2–4). Natural enemies were caught more often (but not significantly so) than their abundance would suggest. This implies that hunting spiders exerted a relatively lower predation pressure on pests than on neutral prey or even on natural enemies. The presence of alternative prey has been shown to disrupt biological control provided by generalist predators (Koss & Snyder, 2005), and in agreement with these findings, our results suggest that hunting spiders can easily switch from pests to neutral (or beneficial) prey hampering the effectiveness of conservation biological control in apple orchards. Although generalist predators (single species or assemblages) can reduce pest numbers (Symondson, Sunderland & Greenstone, 2002), intraguild predation often disrupts their action as biological control agents of herbivores (Rosenheim et al., 1995; Rosenheim, 1998) for example, in the case of unidirectional intraguild predation, where the intermediate predators (e.g., zoophagous bugs, parasitic wasps, hoverflies and lacewings) are more effective at suppressing the target prey (aphids) than the top predators (spiders) (Vance-Chalcraft et al., 2007). As spiders made up almost 45% of the spider-consumed natural enemies (Table S9), it would be difficult to calculate their negative effect on the biological control of pests. Nevertheless, our results suggest that, similar to several multi-enemy systems, hunting spider assemblages in general may often be unable to augment the pest suppression ability of local natural enemies, but instead reduce the overall predation pressure on pests via intraguild predation (Rosenheim, Wilhoit & Armer, 1993; Yasuda & Kimura, 2001; Finke & Denno, 2003, 2004, 2005). However, there are some examples where presence of alternative prey has reduced the intensity of intraguild predation (e.g., Rickers, Langel & Scheu, 2006).

### Specific differences in the diet and in the pest control ability

We found that different spider species exerted different levels of predation on a given prey taxon (Fig. 5; Table S6). Although these trophic interactions strongly depended on both the abundances of predator and prey species and on the season (Figs. S2–S4), the fourth-corner analysis indicated an inherent species-specific prey selection pattern among hunting spiders (Fig. 7; Table S13). This suggests some selectivity in foraging behavior (Nentwig, 1980; Whitney et al., 2018; Eitzinger et al., 2019), but the influence of other species-specific factors such as microhabitat preference (Schmitz & Suttle, 2001), hunting strategy (Schmitz, 2008; Liu et al., 2015; Sanders, Vogel & Knop, 2015) or temporal niche (Morse, 1981; Herberstein & Elgar, 1994; Mezőfi et al., 2019) on the prey composition also cannot be excluded.

We observed relatively high levels of niche overlap, which indicates a functional redundancy (Roubinet et al., 2018) within hunting spiders in the canopy of apple trees (Fig. 6; Tables S11 and S12). Food web specialization was the highest in spring and the lowest in summer (Table 1) and was mainly driven by the prey groups Brachycera, Nematocera and Sternorrhyncha (Figs. S2–S4). Similarly, food web specialization was higher in the early than in the late period of the growing season in barley fields and this variation was suggested to be explained by prey availability dynamics (Roubinet et al., 2018). Species-level specialization was also the highest in spring in four out of six spider groups (Table 1). Overall, *Ph. cespitum* and *Xysticus* spp. showed the highest level of stenophagy and in accordance with that the narrowest niche breadth (Table 1) because *Ph. cespitum* mainly preyed on Nematocera and Sternorrhyncha while the diet of *Xysticus* spp. was comprised mostly of Formicidae and Coleoptera (Fig. 5; Table S6).

The prey composition of *Xysticus* spp. was very different from that of the other spider groups (Fig. 6; Table S11). Brachycera, Nematocera, and Sternorrhyncha were all consumed by *Xysticus* spp. in much lower proportions (16%) than by the other spiders (60–88%) (Fig. 5; Table S6). In contrast to our findings, *Xysticus* spp. was reported to prey intensively on Diptera in hay meadows, *X. cristatus* on aphids in winter wheat and *X. kochi* on thrips pests in greenhouse pepper (Nyffeler & Breene, 1990; Birkhofer et al., 2008; Zrubecz, Toth & Nagy, 2008). Based on these findings it seems that although we found *Xysticus* spp. to be the most stenophagous hunting spider group, it had a high level of plasticity in its use of available resources.

Fourth-corner analysis showed that spider groups discriminated among prey taxa and each spider group had a certain degree of prey preference and avoidance. On a community level, different species of spiders partly complement each other via resource partitioning: if a particular prey group was avoided by one spider group, it was usually preferred by another (Fig. 7; Table S13). For example, compared to the other spiders, Coleoptera and Formicidae prey was rejected by *Ph. cespitum* but preferred by *C. xanthogramma* and *Xysticus* spp. Similar to our results, Michalko & Pekár (2015) found that the *Philodromus* species they studied rejected these two types of prey. The formicid prey of *C. xanthogramma* consisted almost exclusively of winged males as salticids usually refuse dangerous ant workers as prey (Richman & Jackson, 1992; Huseynov, 2005) but *Xysticus* species, as in our case, consume workers frequently (Nyffeler & Breene, 1990; Huseynov, 2014).

It would be expected that spider species within the same guild would exhibit more or less uniform resource utilization patterns (e.g., Luiselli, Akani & Capizzi, 1998; Michalko & Pekár, 2016), but our results do not support this view. In the canopy level of the studied apple orchards, we found marked intraguild differences and interguild similarities in the taxonomic composition of hunting spiders’ prey or in their prey preferences (Figs. 6 and 7). These results agree with Mestre et al. (2013), who reported trophic differences between spider species belonging to the same family. Overall, we found no evidence that hunting guild consistently determines prey composition. Possibly, the guild approach fails to identify finer trophic dynamics, and thus for a more accurate understanding of spider-prey community patterns, the use of (species-specific) functional traits is needed (Fountain-Jones, Baker & Jordan, 2015; Sanders, Vogel & Knop, 2015).

Hunting spiders, among prey designated as pest, most frequently consumed aphids (Table S8). Among hunting spiders, *Ph. cespitum* caught the most aphids in spring (Fig. S2) and possibly contributed to aphid control, especially in the early season by preying upon fundatrices and their larvae (De Roincé et al., 2013; Michalko & Pekár, 2015; Lefebvre et al., 2017). In addition, *Ph. cespitum* also feeds on other pests (Klein, 1988; Wisniewska & Prokopy, 1997; Ghavami, 2008; Michalko et al., 2017). This spider remains active during winter when other predators are dormant, and consequently, it can also reduce, for example, overwintering psyllid populations (Pekár et al., 2015; Petráková et al., 2016). *Ph. cespitum* is among the most abundant hunting spiders in the canopy of apple orchards both in Europe (Bogya, Szinetár & Markó, 1999; Pekár, 1999; Pekár & Kocourek, 2004; Markó et al., 2009) and North America (Miliczky, Horton & Calkins, 2008; Sackett, Buddle & Vincent, 2008), and in our study it consumed the second-highest number of pests (following *Clubiona* spp.), compared to the number of natural enemies it consumed (Fig. 5; Table S6). Based on the above facts, this species could possibly be one of the most effective araneid biological control agents in the canopy of fruit trees in temperate regions, especially in the orchards with reduced use of insecticides (Řezáč, Pekár & Stará, 2010; Michalko & Košulič, 2016; Řezáč, Řezáčová & Heneberg, 2019). Beside *Ph. cespitum*, *Clubiona* spp. (mostly *C. frutetorum*) exerted considerable predation pressure on aphids, especially in autumn (Fig. 5; Fig. S4). There are some examples where spiders reduced aphid infestation by catching aphids immigrating back to the orchard in autumn (Wyss, Niggli & Nentwig, 1995; Cahenzli, Pfiffner & Daniel, 2017) and possibly clubionids also have a high predation potential in this context. Furthermore, according to Madsen, Terkildsen & Toft (2004), the level of predation on aphids by *Clubiona lutescens* Westring is not affected by the presence of alternative prey. Clubionids may also contribute to early season aphid control (Fig. S2; De Roincé et al., 2013) and they may reduce populations of lepidopteran pests by consuming both larvae and adults (Fig. 7; Bogya, 1999). The diet of *Clubiona* spp. had the lowest proportion of natural enemies (Figs. 4 and 5; Table S6), which suggests that clubionids are more compatible with biological control than several other hunting spiders. Meanwhile, *C. xanthogramma* is one of the most common species of spiders in the canopy of pome fruit orchards in Hungary where it can dominate the arboreal spider assemblage (Bogya, Szinetár & Markó, 1999; Bogya, Markó & Szinetár, 2000; Markó & Keresztes, 2014). Due to its high abundance, it exerts strong predation pressure on several prey taxa (Fig. 5; Table S6), but as Markó & Keresztes (2014) previously supposed, in this study, *C. xanthogramma* was found to be a significant intraguild predator of natural enemies, especially spiders (Fig. 5). Beside pests, its diet was comprised of a great number of beneficial prey as well (32% vs. 19%, respectively) and according to the fourth-corner analyses it proved to be the most araneophagic species compared to the other spider taxa examined (Fig. 7; Table S13). Due to its high level of intraguild predation and low abundance in spring, *C. xanthogramma* is possibly not an effective biological control agent. As intraguild predation on spiders is widespread among generalist salticids (Markó & Keresztes, 2014), the arboreal spider assemblage presumably has higher pest suppression potential in apple orchards in northern Europe, where the proportion of salticid spiders in the spider assemblages is lower, than in central or southern Europe where the proportion of salticid spiders is higher (Bogya, Markó & Szinetár, 1999).

Although spiders are characterized as polyphagous predators with a high level of functional redundancy (Figs. 5 and 6; Foelix, 2011; Roubinet et al., 2018), they exert different predation pressure on different arthropod groups (Fig. 5; Figs. S2–S4) and have their own preferences towards certain prey taxa (Fig. 7; Nentwig, 1986), which means that the degree of pest suppression depends on the taxonomic composition of the hunting spider assemblage and on the taxonomic identity of the key pests. In other way, as Birkhofer et al. (2008) suggested, promoting particular species (in our context, *Ph. cespitum* and *Clubiona* spp.) or particular pest-consuming functional groups might be more effective in biological control rather than enhancing predator biodiversity, as the effect of increased diversity is highly context-dependent (Markó & Keresztes, 2014; Michalko et al., 2019).

### Predator–prey size relationships

The size of the prey was strongly related to the size of the hunting spiders, and on average, prey size was 67% that of the spider (see Fig. S1). Analysed separately, there was a significant exponential relationship between the six most abundant spider taxa and their prey, with prey size being 62–77% of predator size (Fig. 8; Table 2). Prey size relates to predator size in both hunting spiders (Nentwig, 1982; Bartos, 2011), web-builders (Brown, 1981; Murakami, 1983), but many other animals (Luiselli, Akani & Capizzi, 1998; Amundsen et al., 2003). This relationship suggests that the size of the prey has an important role in prey selection, especially in active hunters. Hunting spiders have to optimize their energy and nutritional intake while minimizing risk and therefore prefer prey items in the 60–80% range of their own size. However, they regularly captured prey that are larger or smaller than the preferred size (Fig. 8). Furthermore, without taking into account the shape of the spider prosoma, different species of spiders can prefer different prey size (thorax width) relative to their own size (prosoma width): *Ph. cespitum* caught the smallest prey items compared to their body size, followed by *C. xanthogramma*, while clubionids caught the largest prey (Fig. 9; Table 2). We also found a significant difference in niche width with respect to prey size: relative to their own size, *C. xanthogramma* caught prey from the narrowest prey size range, while *Xysticus* spp. caught prey from the broadest size range, suggesting that the size of the prey is not equally important for different hunting spider species (or for different hunting strategies) when choosing prey (Fig. 9). Prey–predator size ratios differed not only between spider groups but also between prey taxa (Fig. 10; Fig. S7). This shows that size and taxonomic identity of the prey are not independent factors. In this study, spiders of the same size caught larger Brachycera than they did Nematocera prey. Finally, the prey–predator size ratio differed between summer and fall. This seasonal difference could be partly explained by prey availability dynamics or by the fact that both the spiders and their prey differed in size between seasons (Table 2; Fig. S5). Nevertheless, the prey–predator size ratios observed were possibly determined not only by the preferences of spiders but to some extent by the available range of prey size in the environment (Tsai, Hsieh & Nakazawa, 2016). Overall, beside taxonomic identity, the size of the prey also matters in prey selection, though its importance may vary depending on the hunting strategy or spider species.

### Ontogenetic niche shifts in *C. xanthogramma* and *Ph. cespitum*

Ontogenetic niche shifts are common in the animal kingdom (Nakazawa, 2015). Such shifts are well documented for example, in aquatic systems (Amundsen et al., 2003), but are largely understudied in spiders. In general, we observed that the diet of *C. xanthogramma* adults included more Coleoptera and Auchenorrhyncha, and fewer Formicidae and Nematocera, while the diet of *Ph. cespitum* adults comprised more Brachycera and Auchenorrhyncha and fewer Nematocera than did the diet of the juveniles (Table S15). However, an ontogenetic niche shift in prey type and size was observed only for *Ph. cespitum*; even though the sesonal occurrence of juveniles overlapped with that of the adults (Figs. 6B and 11). In contrast, *C. xanthogramma* exhibited ontogenetic shift only in prey size, despite little seasonal overlap between the two life stages (Figs. 6D and 11). There was no difference between the life stages in prey–predator size ratio (Fig. S6; Table S14). However, we found an ontogenetic shift in the niche breadth: the adults of *C. xanthogramma* and *Ph. cespitum* preyed upon a wider taxonomic and size range of prey than did their juveniles (Table S14). Bartos (2011) studied the natural prey of another salticid, *Yllenus arenarius* Simon and obtained similar results: the prey size, when standardized relative to spider size, did not differ between life stages, but the trophic niche width increased during the course of the predator’s development. A similar increase in trophic niche width with ontogeny was reported for the philodromid, *Ph. dispar* Walckenaer (Sanders, Vogel & Knop, 2015). In connection to the larger variance in prey size for adults, we found significant relationship between predator and prey size only in juveniles but not in adults (Fig. 11; but see the marginally significant relationship in *Ph. cespitum* adults). In a web-builder spider, *Argiope amoena* L. Koch, Murakami (1983) found a similar relationship: both prey size and prey size range increased with the increase of the prosoma width. A simple explanation for these findings would be that the prey size of spiders is (size-specifically) upper-bounded, but not lower-bounded, and therefore the larger spiders can choose from a wider size and taxonomic range of prey.

## Conclusions

By analyzing a total of 878 hunting spider prey items collected from the canopy of apple trees in apple orchards in Hungary we concluded that (1) although highly polyphagous, arboreal hunting spiders forage selectively and therefore cannot be considered as entirely opportunistic predators. We found that more Brachycera, Nematocera (and possibly Other (non-formicid) Hymenoptera, Lepidoptera and Sternorrhyncha) and less Coleoptera (and possibly Auchenorrhyncha) were consumed by the hunting spider assemblage than would be expected from their abundance in the canopy of apple trees. (2) Hunting spider assemblages consume a large number of pests. However, this beneficial effect is strongly constrained by the high predation levels on natural enemies (intraguild predation) and on neutral insects (propensity to switch from pests to alternative prey). In this study, the hunting spider assemblage showed positive selection for neutral prey, neutral selection for natural enemies and negative selection for pests. (3) In trophic webs, different hunting spider taxa/groups mediate different strengths of trophic effects on different prey taxa, and the web structure changes considerably with the season. (4) The natural prey of hunting spider species is highly overlapped, showing functional redundancy in their predation. (5) Nevertheless, hunting spider species show different trophic niche occupancy, also exhibit a certain level of stenophagy (species-specific prey preference) and select prey by its taxonomic identity and size. (6) The guilds do not determine the preferred or rejected prey types consistently, thus the diet of hunting spiders classified into the same guild can be considerably different. (7) From an economic point of view, *Ph. cespitum* and *Clubiona* spp. were found to be the most effective natural enemies because of their high level of aphid (*Ph. cespitum* and *Clubiona* spp.) and Lepidoptera (*Clubiona* spp.) consumption and low level of intraguild predation. (8) The trophic niche width of *C. xanthogramma* and *Ph. cespitum* increased during ontogeny where adults prey upon a wider taxonomic and size range of arthropods than juveniles. *Ph. cespitum* exhibited an ontogenetic shift in prey type, whereas no such pattern was observed for *C. xanthogramma*.

## Supplemental Information

10.7717/peerj.9334/supp-1 Supplemental Information 1 All prey records of the hunting spiders. For detailed information see the Info sheet of the file.

10.7717/peerj.9334/supp-2 Supplemental Information 2 The comparison of actual and potential prey. For detailed information see the Info sheet of the file.

10.7717/peerj.9334/supp-3 Supplemental Information 3 Supplemental Tables.

10.7717/peerj.9334/supp-4 Supplemental Information 4 Supplemental Figures.

## Additional Information and Declarations

### Data Availability

The following information was supplied regarding data availability: The raw data is available in the Supplemental Files.
