# Supplementary material for: Beyond polyphagy and opportunism: natural prey of hunting spiders in the canopy of apple trees
Source: PeerJ. 2020 Jun 19;8:e9334. doi: 10.7717/peerj.9334 (PMC7307562; doi:10.7717/peerj.9334)
Supplement: Supplemental Information 3 [file peerj-08-9334-s003.pdf]

Mezőfi et al., 2020: Beyond polyphagy and opportunism: natural prey of hunting spiders in the canopy of apple trees.

Supplemental tables

Table S1. **Species composition and abundance of hunting spiders collected in the canopy of apple trees in organic apple orchards**

Taxa	All spiders collected with prey (from 2013 to 2019)		Újfehértó, 2016/17, spiders collected with prey		Újfehértó, 2016/17, suction sampled spiders	
	Abundance	%	Abundance	%	Abundance	%
**Anyphaenidae**	**0**	**0.0%**	**0**	**0.0%**	**1**	**0.3%**
Anyphaena accentuata	0	0.0%	0	0.0%	1	0.3%
**Cheiracanthiidae**	**20**	**2.3%**	**3**	**0.7%**	**8**	**2.6%**
Cheiracanthium cf. *virescens*	1	0.1%	0	0.0%	0	0.0%
Cheiracanthium sp.	19	2.2%	3	0.7%	8	2.6%
**Clubionidae**	**86**	**9.8%**	**57**	**12.6%**	**28**	**9.2%**
Clubiona frutetorum	10	1.1%	5	1.1%	4	1.3%
Clubiona sp.	76	8.7%	52	11.5%	23	7.5%
Porrhoclubiona leucaspis	0	0.0%	0	0.0%	1	0.3%
**Oxyopidae**	**17**	**1.9%**	**5**	**1.1%**	**1**	**0.3%**
Oxyopes ramosus	14	1.6%	4	0.9%	0	0.0%
Oxyopes sp.	3	0.3%	1	0.2%	1	0.3%
**Philodromidae**	**248**	**28.2%**	**76**	**16.8%**	**109**	**35.7%**
Philodromus cespitum	233	26.5%	73	16.2%	9	3.0%
Philodromus longipalpis	4	0.5%	0	0.0%	0	0.0%
Philodromus margaritatus	1	0.1%	0	0.0%	0	0.0%
Philodromus rufus	3	0.3%	2	0.4%	0	0.0%
Philodromus sp. (*aureolus* gr.)	5	0.6%	0	0.0%	100	32.8%
Tibellus oblongus	2	0.2%	1	0.2%	0	0.0%
**Pisauridae**	**2**	**0.2%**	**1**	**0.2%**	**1**	**0.3%**
Pisaura mirabilis	2	0.2%	1	0.2%	1	0.3%
**Salticidae**	**363**	**41.3%**	**240**	**53.1%**	**105**	**34.4%**
Ballus chalybeius	0	0.0%	0	0.0%	1	0.3%
Carrhotus xanthogramma	303	34.5%	214	47.3%	74	24.3%
Evarcha arcuata	2	0.2%	1	0.2%	0	0.0%
Evarcha falcata	2	0.2%	1	0.2%	0	0.0%
Heliophanus auratus	11	1.3%	3	0.7%	4	1.3%
Heliophanus cupreus	17	1.9%	5	1.1%	14	4.6%
Heliophanus sp.	1	0.1%	0	0.0%	0	0.0%
Macaroeris nidicolens	2	0.2%	0	0.0%	0	0.0%
Marpissa muscosa	2	0.2%	0	0.0%	0	0.0%
Pseudicius encarpatus	7	0.8%	4	0.9%	4	1.3%
Salticidae sp.	0	0.0%	0	0.0%	1	0.3%
Salticus scenicus	15	1.7%	12	2.7%	5	1.6%
Salticus sp.	1	0.1%	0	0.0%	2	0.7%
**Thomisidae**	**142**	**16.2%**	**70**	**15.5%**	**52**	**17.0%**
Ebrechtella tricuspidata	53	6.0%	24	5.3%	16	5.2%
Misumena vatia	24	2.7%	15	3.3%	11	3.6%
Runcinia grammica	1	0.1%	0	0.0%	0	0.0%
Synema globosum	4	0.5%	1	0.2%	0	0.0%
Tmarus piger	11	1.3%	8	1.8%	5	1.6%
Xysticus acerbus	3	0.3%	1	0.2%	0	0.0%
Xysticus cristatus	1	0.1%	1	0.2%	0	0.0%
Xysticus kochi	8	0.9%	4	0.9%	0	0.0%
Xysticus lanio	2	0.2%	2	0.4%	0	0.0%
Xysticus sp.	18	2.1%	7	1.5%	18	5.9%
Xysticus (*Spiracme*) *striatipes*	8	0.9%	3	0.7%	1	0.3%
Xysticus ulmi	9	1.0%	4	0.9%	1	0.3%
Sum	878	100.0%	452	100.0%	305	100.0%

Table S2. **Taxonomic composition of the most abundant arboreal hunting spider groups collected in organic apple orchards**

	All spiders collected with prey (from 2013 to 2019)		Újfehértó, 2016/17, spiders collected with prey		Újfehértó, 2016/17, suction sampled spiders	
Taxa	Abundance	%*	Abundance	%*	Abundance	%*
C. xanthogramma	303	34.5%	214	47.4%	74	24.3%
Other salticids	60	6.8%	26	5.8%	31	10.2%
Ph. cespitum	233	26.5%	73	16.2%	109	35.7%
E. tricuspidata	53	6.0%	24	5.3%	16	5.3%
Xysticus spp.	49	5.6%	22	4.9%	20	6.6%
Clubiona spp.	86	9.8%	57	12.6%	28	9.2%
Sum	784	89.3%	416	92.0%	278	91.2%

*Relative to the whole arboreal hunting spider assemblage

Table S3. **Number of hunting spiders collected monthly by spider groups**

Month/Taxa	*C. xanthogramma*	Other salticids	*Ph. cespitum*	*E. tricuspidata*	*Xysticus* spp.	*Clubiona* spp.*
**All collected spiders with prey**						
April	9	9	67	18	13	15
May	36	22	106	11	4	14
June	25	7	37	4	4	4
July	126	9	16	5	15	5
August	38	5	4	7	5	9
September	52	4	2	8	8	26
October	17	4	1	0	0	12
**Újfehértó, 2016/17, collected spiders with prey**						
April	4	4	28	8	7	9
May	18	6	17	6	1	4
June	23	3	11	2	1	2
July	110	6	14	4	8	5
August	27	2	1	3	5	7
September	15	1	1	1	0	17
October	17	4	1	0	0	12
**Újfehértó, 2016/17, suction sampled spiders**						
April	3	1	39	5	0	0
May	3	10	30	0	0	1
June	5	3	19	0	3	4
July	23	5	4	0	11	3
August	16	9	6	7	4	10
September	13	2	2	2	1	8
October	11	1	9	2	1	2

Two matrices were compared with Mantel test

*One record collected in March is not indicated in this Table.

Table S4. **Number of the actual and potential prey items of arboreal hunting spiders by prey groups and months**

Month/Taxa	Araneae	Coleoptera	Lepidoptera	Formicidae [a]	Other Hymenoptera	Brachycera	Nematocera	Auchenorrhyncha	Heteroptera	Sternorrhyncha [b]	Other [c]
**All collected spiders - Actual prey**											
April	6	8	4	6	1	15	58	0	4	42	3
May	14	23	1	7	2	40	80	1	2	46	2
June	1	1	0	6	0	34	12	2	2	35	2
July	15	3	4	33	8	36	26	8	4	37	12
August	6	1	2	1	1	26	8	4	14	7	0
September	14	3	4	11	8	15	14	13	14	20	4
October	1	0	0	4	0	3	7	2	2	20	0
**Újfehértó, 2016/17, - Actual prey**											
April	2	5	2	3	1	4	26	0	0	24	1
May	3	10	0	6	1	18	13	1	0	7	1
June	0	1	0	3	0	12	8	0	0	24	0
July	10	1	3	26	8	32	25	7	2	30	10
August	5	1	2	0	1	19	6	4	4	5	0
September	2	0	2	2	4	6	8	4	4	4	0
October	1	0	0	3	0	2	6	2	2	20	0
**Újfehértó, 2016/17, suction sampled - Potential prey**											
April	61	85	4	6	7	50	134	25	16	14	29
May	59	998	4	425	19	141	143	10	14	170	11
June	48	149	18	142	9	162	39	17	22	5996	25
July	62	52	8	42	15	39	75	136	37	16	15
August	56	30	13	24	6	18	14	195	62	5	13
September	45	53	8	5	12	47	62	248	137	5	17
October	65	49	3	3	21	27	260	246	25	70	28

Two matrices were compared with Mantel test

[a]One record collected in March is not indicated in this Table.

[b]Two records collected in March is not indicated in this Table.

[c]Acari, Ephemeroptera, Neuroptera, Psocoptera, Thysanoptera, Trichoptera, while in case of Potential prey, Orthoptera, Raphidioptera and Indet were also included.

Table S5. **Prey items of the six most abundant arboreal hunting spider groups**

Prey/Predator	*C. xanthogramma*	Other salticids	*Ph. cespitum*	*E. tricuspidata*	*Xysticus* spp.	*Clubiona* spp.
**Újfehértó, 2016-2017**						
Araneae	14	2	2	1	1	2
Coleoptera	6	1	1	2	5	0
Lepidoptera	4	0	0	1	0	3
Formicidae	22	0	0	0	8	3
Other Hymenoptera	6	1	1	3	0	1
Brachycera	60	7	8	5	3	5
Nematocera	26	7	30	8	1	13
Auchenorrhyncha	9	0	4	0	1	4
Heteroptera	6	0	1	0	3	2
Sternorrhyncha	53	8	22	4	0	24
Other prey*	8	0	4	0	0	0
**All sites and sampling dates except 2016-2017, Újfehértó**						
Araneae	14	4	8	1	1	3
Coleoptera	5	1	0	1	8	0
Lepidoptera	0	1	0	1	0	1
Formicidae	3	0	0	1	12	0
Other Hymenoptera	3	2	0	0	0	0
Brachycera	16	6	28	6	0	8
Nematocera	10	3	75	9	3	4
Auchenorrhyncha	6	1	1	0	0	1
Heteroptera	9	4	3	6	2	1
Sternorrhyncha	17	11	42	4	1	11
Other prey*	6	1	3	0	0	0

Two matrices were compared with Mantel test

*Acari, Ephemeroptera, Neuroptera, Psocoptera, Thysanoptera, Trichoptera

Table S6. **Raw data of the trophic web (Fig. 5 based on abundance data).**
Taxonomic and economic composition of natural prey of hunting spider groups for all sites and years

Prey/Predator	*C. xanthogramma*		Other salticids		*Ph. cespitum*		*E. tricuspidata*		*Xysticus* spp.		*Clubiona* spp.	
Acari	0	0.0%	0	0.0%	3	1.3%	0	0.0%	0	0.0%	0	0.0%
Araneae	28	9.2%	6	10.0%	10	4.3%	2	3.8%	2	4.1%	5	5.8%
Coleoptera	11	3.6%	2	3.3%	1	0.4%	3	5.7%	13	26.5%	0	0.0%
Lepidoptera	4	1.3%	1	1.7%	0	0.0%	2	3.8%	0	0.0%	4	4.7%
Formicidae	25	8.3%	0	0.0%	0	0.0%	1	1.9%	20	40.8%	3	3.5%
Other Hymenoptera	9	3.0%	3	5.0%	1	0.4%	3	5.7%	0	0.0%	1	1.2%
Brachycera	76	25.1%	13	21.7%	36	15.5%	11	20.8%	3	6.1%	13	15.1%
Nematocera	36	11.9%	10	16.7%	105	45.1%	17	32.1%	4	8.2%	17	19.8%
Auchenorrhyncha	15	5.0%	1	1.7%	5	2.1%	0	0.0%	1	2.0%	5	5.8%
Heteroptera	15	5.0%	4	6.7%	4	1.7%	6	11.3%	5	10.2%	3	3.5%
Sternorrhyncha	70	23.1%	19	31.7%	64	27.5%	8	15.1%	1	2.0%	35	40.7%
Ephemeroptera	2	0.7%	0	0.0%	0	0.0%	0	0.0%	0	0.0%	0	0.0%
Neuroptera	4	1.3%	1	1.7%	3	1.3%	0	0.0%	0	0.0%	0	0.0%
Psocoptera	5	1.7%	0	0.0%	0	0.0%	0	0.0%	0	0.0%	0	0.0%
Thysanoptera	2	0.7%	0	0.0%	1	0.4%	0	0.0%	0	0.0%	0	0.0%
Trichoptera	1	0.3%	0	0.0%	0	0.0%	0	0.0%	0	0.0%	0	0.0%
Natural enemy	58	19.1%	12	20.0%	21	9.0%	10	18.9%	6	12.2%	9	10.5%
Neutral	149	49.2%	27	45.0%	143	61.4%	31	58.5%	31	63.3%	34	39.5%
Pest	96	31.7%	21	35.0%	69	29.6%	12	22.6%	12	24.5%	43	50.0%
Sum:	303	100%	60	100%	233	100%	53	100%	49	100%	86	100%

Table S7. **Taxonomic composition of actual and potential prey items of arboreal hunting spiders collected in apple orchards**

	All collected spiders - Actual prey		Újfehértó, 2016/17, - Actual prey		Újfehértó, 2016/17, suction sampled - Potential prey	
Taxa	Abundance	%	Abundance	%	Abundance	%
Acari	3	0.3%	1	0.2%	14	0.1%
Araneae	57	6.5%	23	5.1%	396	3.5%
Coleoptera	39	4.4%	18	4.0%	1416	12.4%
Lepidoptera	15	1.7%	9	2.0%	58	0.5%
Formicidae	69	7.9%	44	9.7%	647	5.7%
Other Hymenoptera	20	2.3%	15	3.3%	89	0.8%
Brachycera	169	19.2%	93	20.6%	484	4.2%
Nematocera	205	23.3%	92	20.4%	727	6.4%
Auchenorrhyncha	30	3.4%	18	4.0%	877	7.7%
Heteroptera	42	4.8%	12	2.7%	313	2.7%
Sternorrhyncha	209	23.8%	116	25.7%	6276	55.0%
Ephemeroptera	3	0.3%	2	0.4%	1	0.0%
Neuroptera	8	0.9%	5	1.1%	92	0.8%
Psocoptera	5	0.6%	2	0.4%	15	0.1%
Thysanoptera	3	0.3%	2	0.4%	2	0.0%
Trichoptera	1	0.1%	0	0.0%	1	0.0%
Other*	0	0.0%	0	0.0%	13	0.1%
Sum	878	100.0%	452	100.0%	11421	100.0%

*Orthoptera, Raphidioptera, Indet

Table S8. **Taxonomic composition of actual prey items of arboreal hunting spiders collected in apple orchards – pests.** All sites and years are included.

Taxa	Abundance	%	Example
**Auchenorrhyncha**	**18**	**6.5%**	
Cicadellidae	2	0.7%	*Empoasca* sp.
Flatidae	16	5.8%	*Metcalfa pruinosa*
**Coleoptera**	**30**	**10.8%**	
Cerambycidae	1	0.4%	*Pogonocherus hispidus*
Curculionidae	29	10.5%	*Phyllobius betulinus*, *Ph. oblongus*, *Ph. virideaeris*, *Rhamphus* sp.
**Heteroptera**	**6**	**2.2%**	
Tingidae	6	2.2%	*Stephanitis pyri*
**Lepidoptera**	**14**	**5.1%**	
Arctiidae	1	0.4%	*Hyphantria cunea*
Gracillariidae	1	0.4%	*Phyllonorycter blancardella*
Indet leaf miners	2	0.7%	
Sesiidae	1	0.4%	*Synanthedon myopaeformis*
Tortricidae	9	3.2%	cf. *Recurvaria* sp.
**Sternorrhyncha**	**209**	**75.5%**	
Aphididae	198	71.5%	*Aphis pomi*, *Dysaphis devecta*, *D. plantaginea*
Psyllidae	11	4.0%	*Cacopsylla mali*, *C. melanoneura*
Sum	277	100.0%	

Table S9. **Taxonomic composition of actual prey items of arboreal hunting spiders collected in apple orchards – natural enemies.** All sites and years are included.

Taxa	Abundance	%	Example
**Acari**	**2**	**1.6%**	Trombidiidae sp.
**Araneae**	**57**	**44.5%**	
Araneidae	5	3.9%	*Cyclosa oculata*, *Mangora acalypha*
Cheiracanthiidae	1	0.8%	*Cheiracanthium* sp.
Clubionidae	5	3.9%	*Clubiona frutetorum*
Linyphiidae	3	2.3%	*Erigone dentipalpis*, *Gongylidiellum murcidum*, *Porrhomma microphthalmum*
Lycosidae	1	0.8%	
Philodromidae	9	7.0%	*Philodromus cespitum*
Salticidae	13	10.2%	*Carrhotus xanthogramma*, *Heliophanus auratus*, *H. cupreus*, *Salticus* sp.
Theridiidae	5	3.9%	*Dipoena melanogaster*, *Lasaeola prona*, *Theridion* sp.
Thomisidae	3	2.3%	*Ebrechtella tricuspidata*, *Ozyptila* sp., *Xysticus* sp.
Indet	12	9.4%	
**Brachycera**	**14**	**10.9%**	
Syrphidae	14	10.9%	*Episyrphus balteatus*, *Eupeodes corollae*
**Coleoptera**	**4**	**3.1%**	
Carabidae	1	0.8%	
Coccinellidae	2	1.6%	*Adalia decempunctata*, *Stethorus* sp.
Staphylinidae	1	0.8%	
**Heteroptera**	**26**	**20.3%**	
Anthocoridae	6	4.7%	*Orius* sp.
Miridae	20	15.6%	*Agnocoris* sp., *Campylomma verbasci*, *Deraeocoris ruber*, *Pilophorus perplexus*
**Neuroptera**	**8**	**6.3%**	Chrysopidae sp., Hemerobiidae sp.
**Hymenoptera**	**17**	**13.3%**	Hymenoptera Parasitica spp.
Sum	128	100.0%	

Table S10. **Ivlev's electivity indices based on the actual and potential prey of the arboreal hunting spider assemblage, Újfehértó, Hungary, 2016-2017.** Comparison of the proportion of each prey group in the actual and potential prey based on model contrasts.

Taxa/Economic category	Ivlev's index	*P*[a]
Araneae	0.0568	0.7185
Coleoptera	-0.5472	0.0003
Lepidoptera	0.4897	0.0765
Formicidae	0.2080	0.1415
Other Hymenoptera	0.4986	0.0914
Brachycera	0.5789	0.0023
Nematocera	0.4018	0.0246
Auchenorrhyncha	-0.3143	0.0534
Heteroptera	-0.1067	0.2658
Sternorrhyncha	0.0191	0.0814
Other prey[b]	0.0295	0.8313
Natural enemy	0.0908	0.8941
Neutral	0.4084	0.0017
Pest	-0.3050	0.0023

[a]GLMM-b contrasts

[b]Acari, Ephemeroptera, Neuroptera, Psocoptera, Thysanoptera, Trichoptera

Table S11. **Similarity (trophic niche overlap index) between the prey of the six hunting spider groups**

	C. xanthogramma	Other salticids	*Ph. cespitum*	*E. tricuspidata*	*Xysticus* spp.
	Niche overlap based on prey taxonomic composition				
Other salticids	0.815	*	*	*	*
Ph. cespitum	**0.612**	**0.694**	*	*	*
E. tricuspidata	**0.663**	0.730	0.690	*	*
Xysticus spp.	**0.393**	**0.321**	**0.246**	**0.379**	*
Clubiona spp.	**0.703**	0.773	**0.709**	0.641	**0.294**
	Niche overlap based on prey size				
Other salticids	0.887	*	*	*	*
Ph. cespitum	0.833	0.840	*	*	*
E. tricuspidata	0.883	0.832	0.858	*	*
Xysticus spp.	0.898	0.806	0.764	0.893	*
Clubiona spp.	0.800	0.861	0.777	0.725	0.719
	Overall niche overlap				
Other salticids	0.851	*	*	*	*
Ph. cespitum	**0.722**	**0.767**	*	*	*
E. tricuspidata	**0.773**	0.781	0.774	*	*
Xysticus spp.	**0.645**	**0.563**	**0.505**	**0.636**	*
Clubiona spp.	**0.751**	0.817	**0.743**	**0.683**	**0.506**

0 index value means no overlap, whereas values close to 1 reflect similar resource utilization spectra.

Species pairs occupying statistically different niches, as identified by null model tests, are indicated in bold (adjusted α = 0.0033).

Table S12. **Similarity (trophic niche overlap index) between the prey of the six hunting spider groups.**
C. xanthogramma and *Ph. cespitum* were split to juveniles and adults

	C. xanthogramma juveniles	*C. xanthogramma* adults	Other salticids	*Ph. cespitum* juveniles	*Ph. cespitum* adults	*E. tricuspidata*	*Xysticus* spp.
	Niche overlap based on prey taxonomic composition						
C. xanthogramma adults	0.784	*	*	*	*	*	*
Other salticids	0.801	0.783	*	*	*	*	*
Ph. cespitum juveniles	**0.542**	**0.465**	**0.588**	*	*	*	*
Ph. cespitum adults	0.737	0.702	0.804	**0.707**	*	*	*
E. tricuspidata	**0.637**	0.655	0.730	**0.622**	0.723	*	*
Xysticus spp.	**0.392**	**0.382**	**0.321**	**0.216**	**0.255**	**0.379**	*
Clubiona spp.	**0.707**	**0.647**	0.773	**0.604**	0.788	0.641	**0.294**
	Niche overlap based on prey size						
C. xanthogramma adults	**0.661**	*	*	*	*	*	*
Other salticids	0.912	**0.712**	*	*	*	*	*
Ph. cespitum juveniles	0.825	**0.629**	0.814	*	*	*	*
Ph. cespitum adults	0.837	**0.738**	0.852	0.855	*	*	*
E. tricuspidata	0.806	0.755	0.832	0.805	0.929	*	*
Xysticus spp.	0.795	0.785	0.806	**0.726**	0.831	0.893	*
Clubiona spp.	0.888	**0.598**	0.861	0.786	0.762	0.725	0.719
	Overall niche overlap						
C. xanthogramma adults	**0.723**	*	*	*	*	*	*
Other salticids	0.856	**0.748**	*	*	*	*	*
Ph. cespitum juveniles	**0.684**	**0.547**	**0.701**	*	*	*	*
Ph. cespitum adults	**0.787**	**0.720**	0.828	**0.781**	*	*	*
E. tricuspidata	**0.722**	**0.705**	0.781	**0.714**	0.826	*	*
Xysticus spp.	**0.594**	**0.584**	**0.563**	**0.471**	**0.543**	**0.636**	*
Clubiona spp.	0.798	**0.623**	0.817	**0.695**	0.775	**0.683**	**0.506**

0 index value means no overlap, whereas values close to 1 reflect similar resource utilization spectra.
Species pairs occupying statistically different niches, as identified by null model tests, are indicated in bold (adjusted $\alpha = 0.0018$).

Table S13. **Coefficients of prey taxa vs. spider groups and the environmental predictors (fourth-corner model)**

Prey taxa / Predictors	*C. xanthogramma*	Other salticids	*Ph. cespitum*	*E. tricuspidata*	*Xysticus* spp.	*Clubiona* spp.	Spring	Summer	Fall
Araneae	0.013	0.007	0	-0.046	0	0	0.069	0	0
Coleoptera	0.030	0	-0.157	0	0.265	-0.094	0.321	-0.030	0
Lepidoptera	-0.051	0	-0.095	0.046	0	0.087	0	0	0
Formicidae	0.039	-0.113	-0.188	0	0.303	0	-0.029	0.121	0
Other Hymenoptera	0	0.069	0	0.089	0	0	-0.035	0	0.095
Brachycera	0	0	0.029	0	-0.052	0	0	0	-0.117
Nematocera	-0.058	-0.039	0.115	0.021	-0.031	0	0.152	0	0
Auchenorrhyncha	0	0	0.072	-0.035	0	0.025	-0.164	0	0.111
Heteroptera	-0.030	0	-0.005	0.076	0.107	-0.012	-0.084	0	0.012
Sternorrhyncha	0	0.036	0.093	-0.029	-0.136	0.089	0.046	0	-0.015
Other prey*	0.044	0	0.102	0	0	-0.032	0	0.040	0

*Acari, Ephemeroptera, Neuroptera, Psocoptera, Thysanoptera, Trichoptera

Coefficients (in absolute value) ≥ 0.03 are highlighted in red (positive associations) or in blue (negative associations).

Table S14. **Trophic niche widths (Levins' *B*), predator-prey size data and niche widths with respect to size (s^2) for different life stages of *C. xanthogramma* and *Ph. cespitum***

	C. xanthogramma juveniles	*C. xanthogramma* adults	*Ph. cespitum* juveniles	*Ph. cespitum* adults
B	5.851	6.762	2.509	4.322
Spider prosoma width (mm), mean (SD)	1.48 (0.30)	2.48 (0.23)	1.24 (0.24)	1.87 (0.30)
Prey thorax width (mm), mean (SD)	0.97 (0.51)	1.52 (0.69)	0.81 (0.52)	1.09 (0.65)
Thorax-prosoma ratio, mean (SD)	0.65 (0.27)	0.61 (0.28)	0.64 (0.38)	0.58 (0.33)
s^2 absolute predator size	0.09 A	0.05 A	0.06 a	0.09 b
s^2 absolute prey size	0.26 A	0.46 B	0.23 a	0.40 b
s^2 prey-predator size ratio	0.07 A	0.08 A	0.15 a	0.11 a

Different capital letters indicate significant differences in *C. xanthogramma* and different lowercase letters indicate significant differences in *Ph. cespitum* at $P < 0.05$ level.

Table S15. **Natural prey of juveniles and adults (subadults + adults) of *Carrhotus xanthogramma* and *Philodromus cespitum***

	Carrhotus xanthogramma				*Philodromus cespitum*			
	Abundance		%		Abundance		%	
	Juveniles	Adults	Juveniles	Adults	Juveniles	Adults	Juveniles	Adults
Acari	0	0	0.0%	0.0%	1	2	0.8%	2.0%
Araneae	17	11	9.2%	9.6%	5	5	3.8%	5.1%
Coleoptera	1	10	0.5%	8.8%	1	0	0.8%	0.0%
Lepidoptera	3	1	1.6%	0.9%	0	0	0.0%	0.0%
Formicidae	22	2	11.9%	1.8%	0	0	0.0%	0.0%
Other Hymenoptera	4	4	2.2%	3.5%	0	1	0.0%	1.0%
Brachycera	48	28	25.9%	24.6%	13	23	9.8%	23.5%
Nematocera	25	10	13.5%	8.8%	75	28	56.4%	28.6%
Auchenorrhyncha	4	11	2.2%	9.6%	0	5	0.0%	5.1%
Heteroptera	8	6	4.3%	5.3%	1	3	0.8%	3.1%
Sternorrhyncha	45	25	24.3%	21.9%	35	29	26.3%	29.6%
Ephemeroptera	2	0	1.1%	0.0%	0	0	0.0%	0.0%
Neuroptera	2	2	1.1%	1.8%	1	2	0.8%	2.0%
Psocoptera	2	3	1.1%	2.6%	0	0	0.0%	0.0%
Thysanoptera	2	0	1.1%	0.0%	1	0	0.8%	0.0%
Trichoptera	0	1	0.0%	0.9%	0	0	0.0%	0.0%
Sum:	185	114	100.0%	100.0%	133	98	100.0%	100.0%
