# Supplementary material for: Beyond polyphagy and opportunism: natural prey of hunting spiders in the canopy of apple trees
Source: PeerJ. 2020 Jun 19;8:e9334. doi: 10.7717/peerj.9334 (PMC7307562; doi:10.7717/peerj.9334)
Supplement: Supplemental Information 4 [file peerj-08-9334-s004.pdf]

Mezőfi et al., 2020: Beyond polyphagy and opportunism: natural prey of hunting spiders in the canopy of apple trees.

Supplemental figures

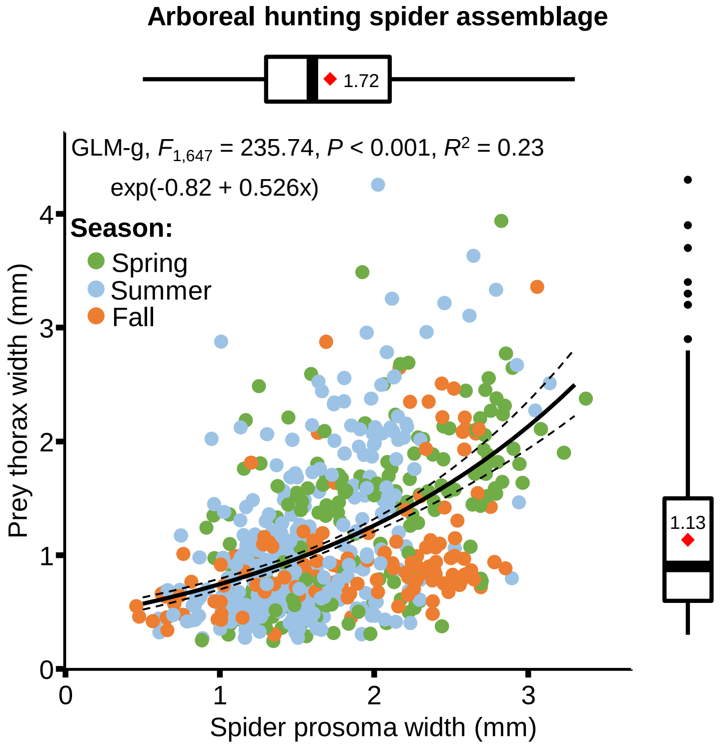

Figure S1: Relationship between spider prosoma and prey thorax widths (jittered, N=649) for the arboreal hunting spider assemblage.

On the marginal boxplots red squares indicate the mean values.

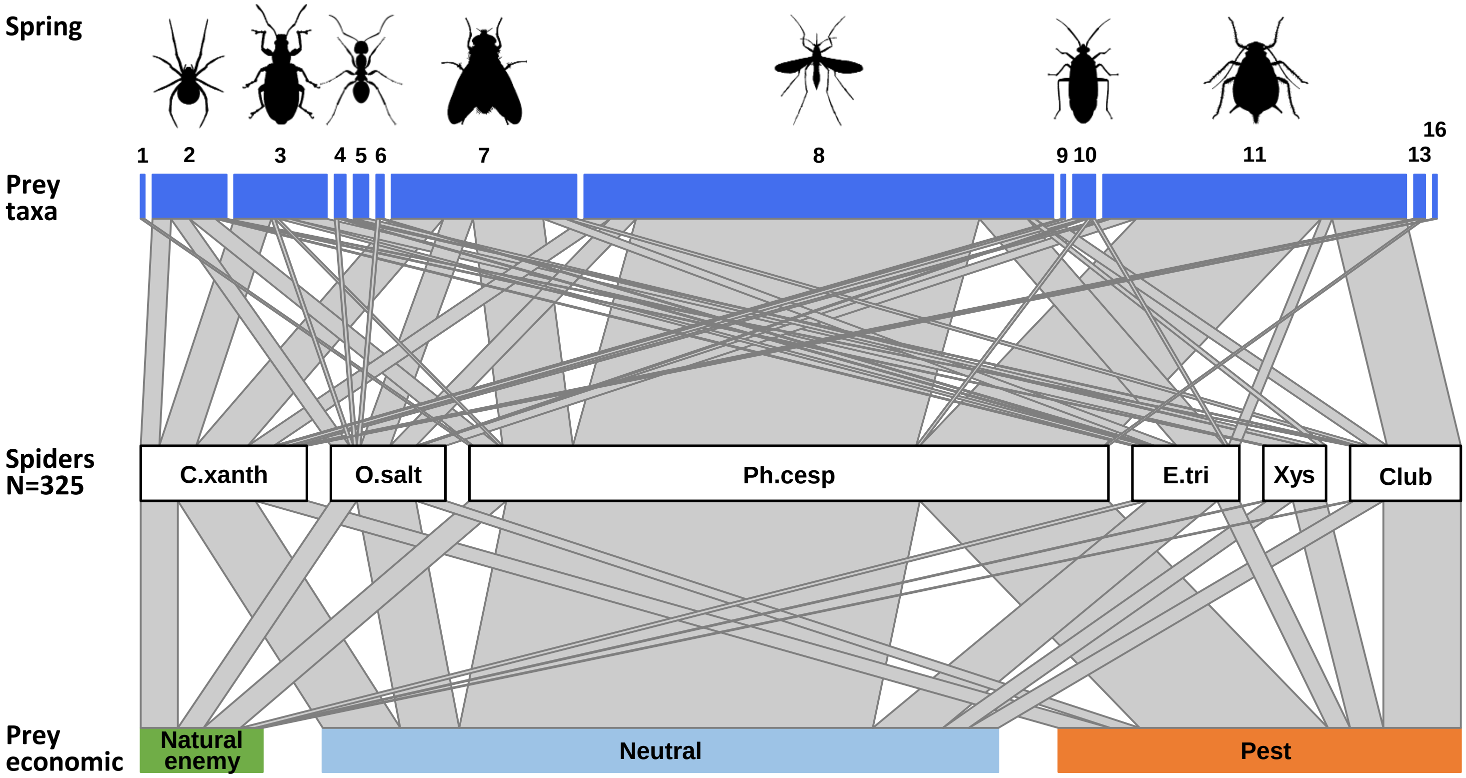

Figure S2: Spring aspect (N=325); throphic interactions between the most abundant hunting spider groups and the arthropod community in the canopy of apple trees in spring. The middle bars represent spider groups and upper and bottom bars represent the spiders' prey divided taxonomically and according their economic status. The width of the links between the trophic levels depict the frequency of interactions and bar widths indicate the relative abundance of each category. Numbers refer to following prey taxa: **1** Acari, **2** Araneae, **3** Coleoptera, **4** Lepidoptera, **5** Formicidae, **6** Other Hymenoptera, **7** Brachycera, **8** Nematocera, **9** Auchenorrhyncha, **10** Heteroptera, **11** Sternorrhyncha, **13** Neuroptera, **16** Trichoptera; Spiders: **C.xanth** = *Carrhotus xanthogramma*, **O.salt** = Other salticids, **Ph.cesp** = *Philodromus cespitum*, **E.tri** = *Ebrechtella tricuspidata*, **Xys** = *Xysticus* spp., **Club** = *Clubiona* spp.

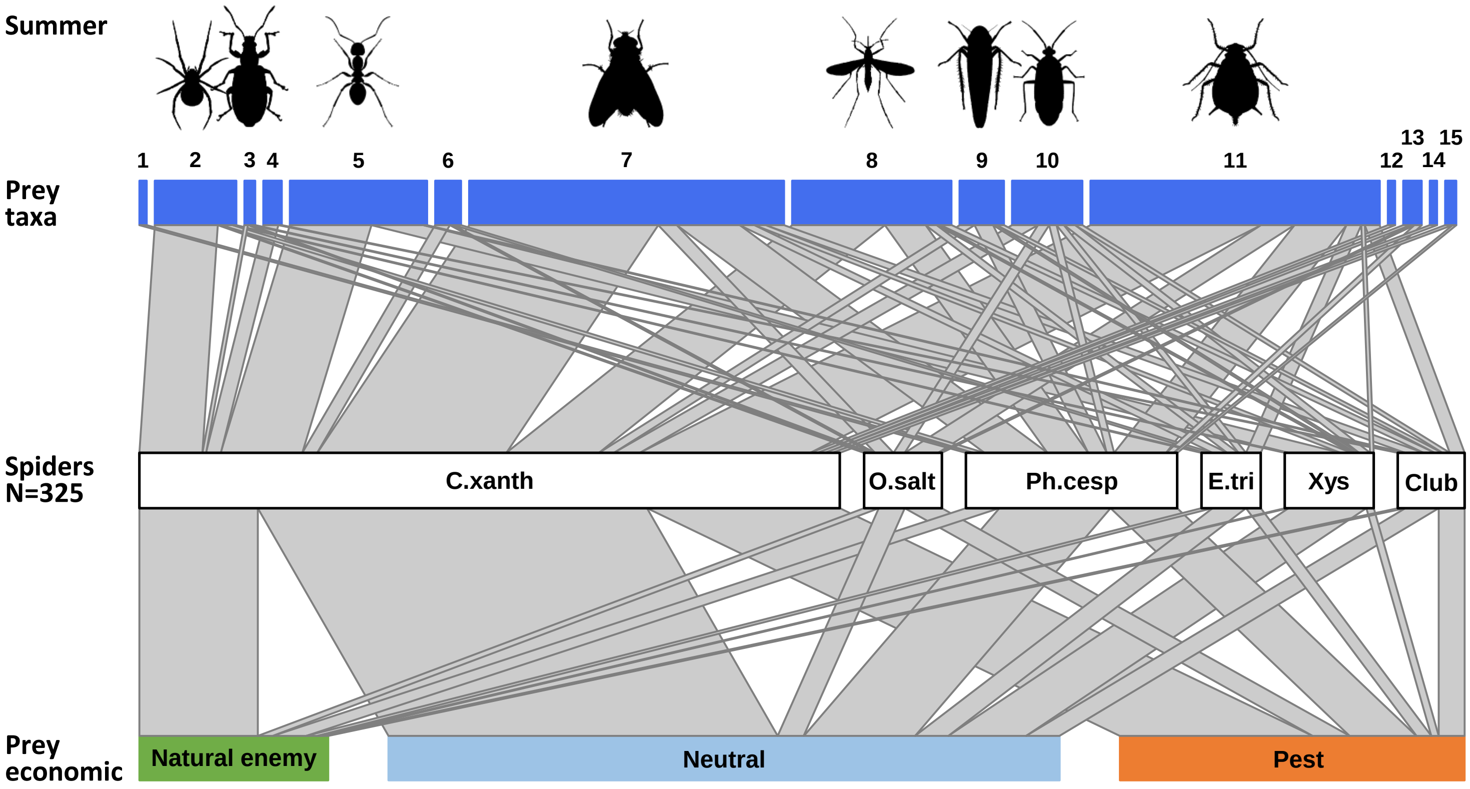

Figure S3: Summer aspect (N=325); throphic interactions between the most abundant hunting spider groups and the arthropod community in the canopy of apple trees in summer.

The middle bars represent spider groups and upper and bottom bars represent the spiders' prey divided taxonomically and according their economic status. The width of the links between the trophic levels depict the frequency of interactions and bar widths indicate the relative abundance of each category. Numbers refer to following prey taxa: **1** Acari, **2** Araneae, **3** Coleoptera, **4** Lepidoptera, **5** Formicidae, **6** Other Hymenoptera, **7** Brachycera, **8** Nematocera, **9** Auchenorrhyncha, **10** Heteroptera, **11** Sternorrhyncha, **12** Ephemeroptera, **13** Neuroptera, **14** Psocoptera, **15** Thysanoptera; Spiders: **C.xanth** = *Carrhotus xanthogramma*, **O.salt** = Other salticids, **Ph.cesp** = *Philodromus cespitum,* **E.tri** = *Ebrechtella tricuspidata*, **Xys** = *Xysticus* spp., **Club** = *Clubiona* spp.

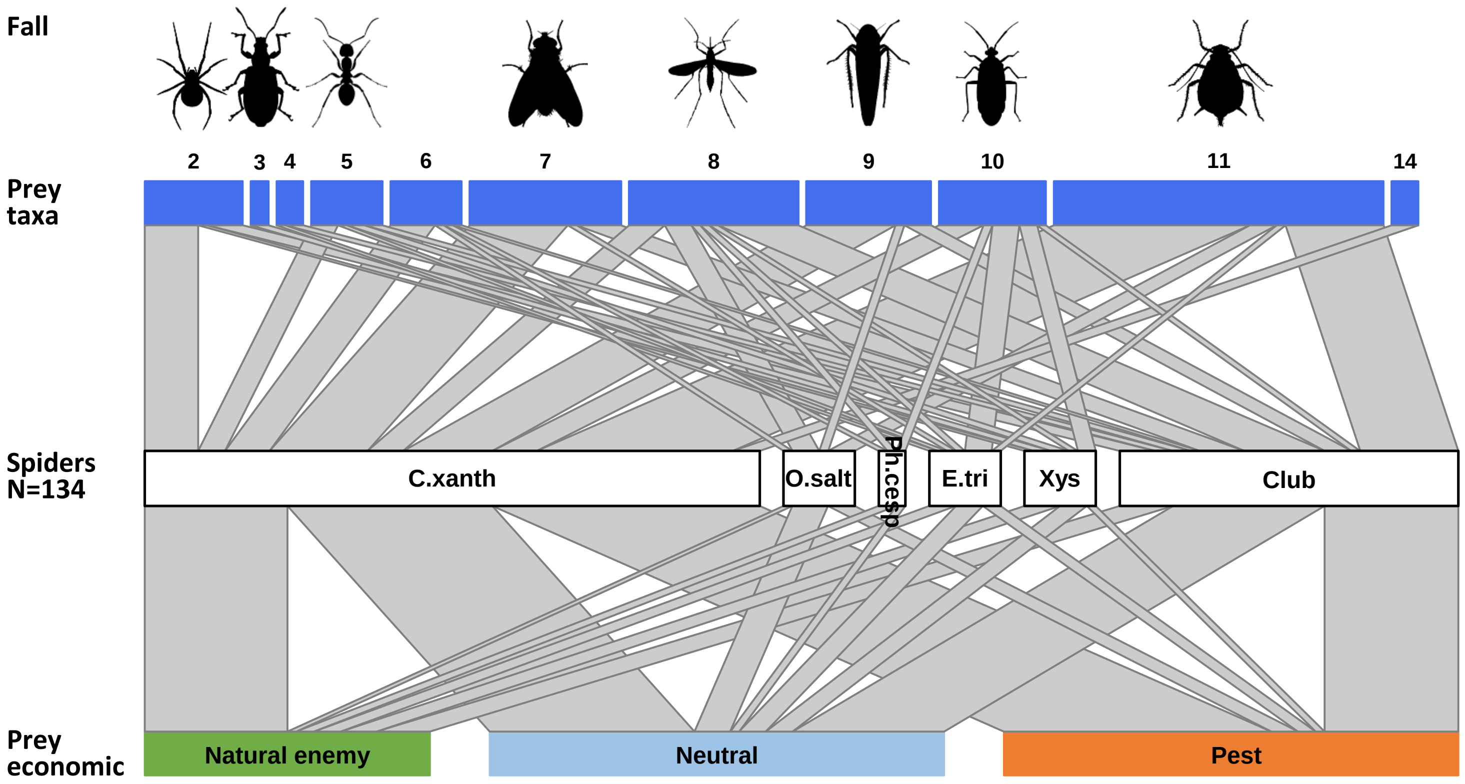

Figure S4: Fall aspect (N=134); throphic interactions between the most abundant hunting spider groups and the arthropod community in the canopy of apple trees in fall.
The middle bars represent spider groups and upper and bottom bars represent the spiders' prey divided taxonomically and according their economic status. The width of the links between the trophic levels depict the frequency of interactions and bar widths indicate the relative abundance of each category. Numbers refer to following prey taxa: **2** Araneae, **3** Coleoptera, **4** Lepidoptera, **5** Formicidae, **6** Other Hymenoptera, **7** Brachycera, **8** Nematocera, **9** Auchenorrhyncha, **10** Heteroptera, **11** Sternorrhyncha, **14** Psocoptera; Spiders: **C.xanth** = *Carrhotus xanthogramma*, **O.salt** = Other salticids, **Ph.cesp** = *Philodromus cespitum,* **E.tri** = *Ebrechtella tricuspidata*, **Xys** = *Xysticus* spp., **Club** = *Clubiona* spp.

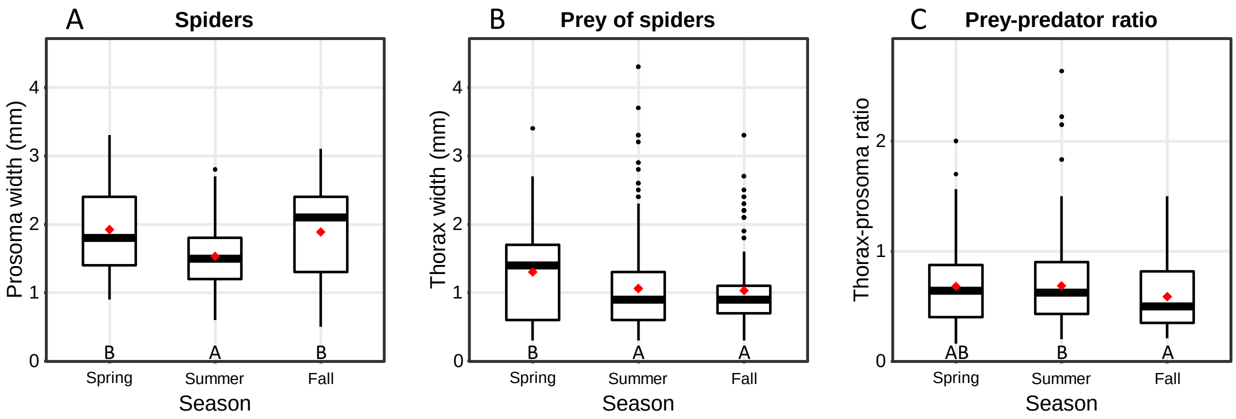

Figure S5: Seasonal variation in spider prosoma width (A), prey thorax width (B) and the prey thorax and spider prosoma ratio (C).

On the boxplots red squares indicate the mean values. Different capital letters indicate significant differences between the seasons at *P* < 0.05 level.

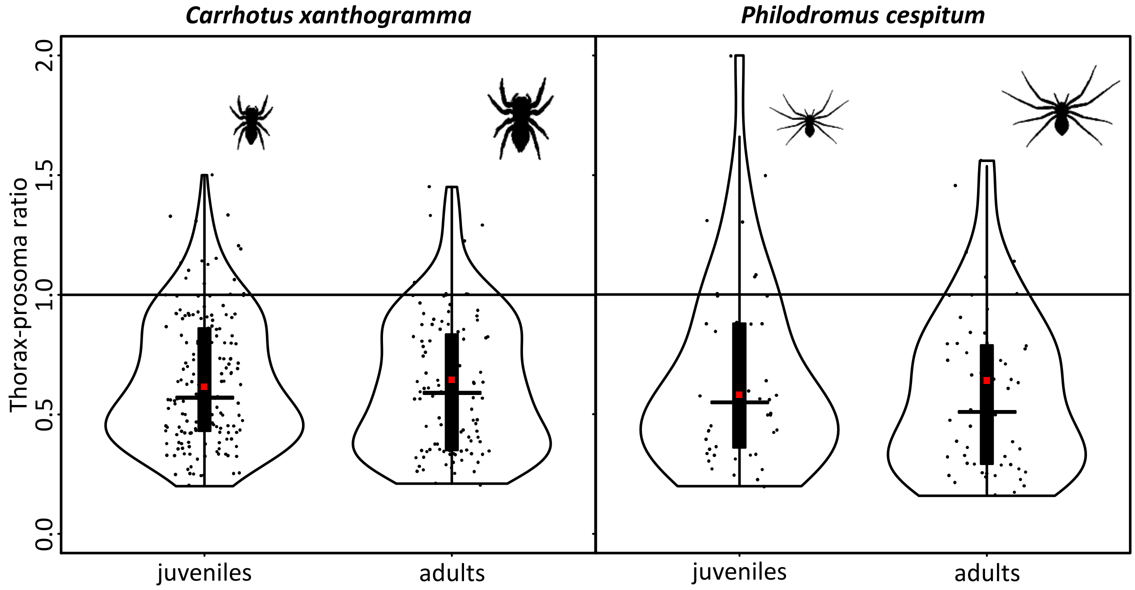

Figure S6: Prey thorax and spider prosoma ratios (jittered) for the juveniles and adults of *C. xanthogramma* and *Ph. cespitum*.

Note that adults comprise both the subadult and adult individuals. Red square – mean; black horizontal solid line – median; black vertical rectangle – interquartile range.

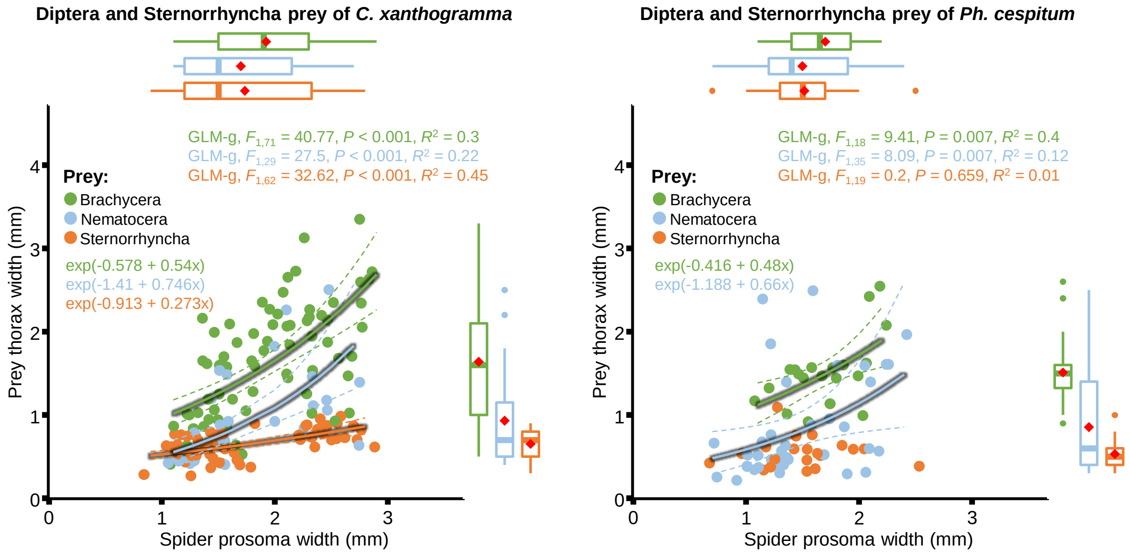

Figure S7: Relationship between spider prosoma and prey thorax widths (jittered) for the two most abundant arboreal hunting spider species, *Carrhotus xanthogramma* and *Philodromus cespitum*, and their main prey taxa (Brachycera, Nematocera and Sternorrhyncha) in apple orchards.

On the marginal boxplots red squares indicate the mean values.
